# Supplementary material for: Chemical labelling for visualizing native AMPA receptors in live neurons
Source: Nat Commun. 2017 Apr 7;8:14850. doi: 10.1038/ncomms14850 (PMC5385570; doi:10.1038/ncomms14850)
Supplement: Supplementary Information — Supplementary Figures, Supplementary Table, Supplementary Methods and Supplementary References [file ncomms14850-s1.pdf]

Supplementary Figures

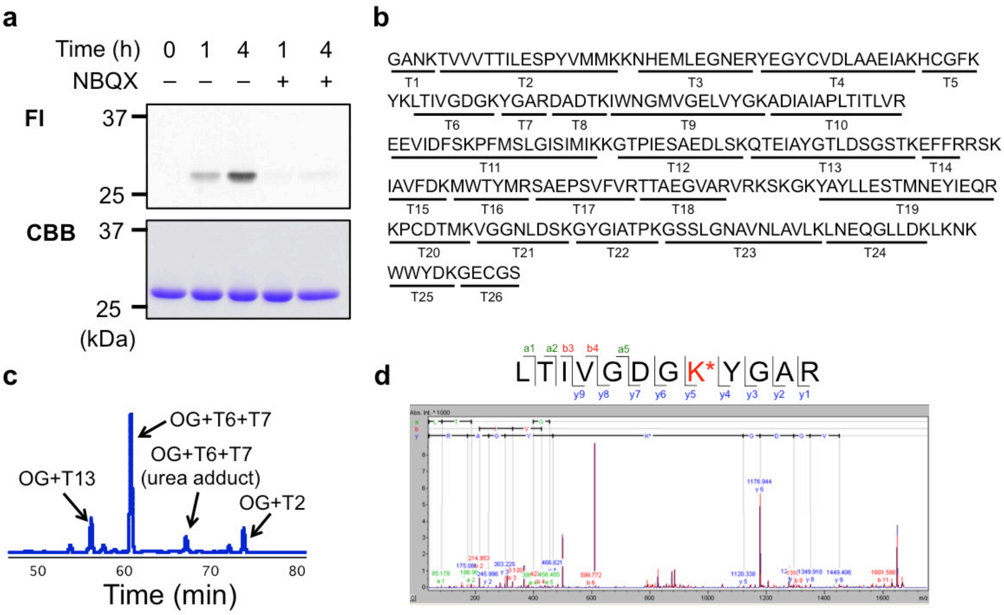

Supplementary Figure 1 | Identification of labeling site of CAM2(OG) to AMPARs using a recombinant of the ligand binding domain of GluA2. (**a**) Chemical labeling of the ligand binding domain of GluA2(S1S2J) with **CAM2(OG)**. Three μM of S1S2J was incubated with 10 μM of **CAM2(OG)** in the buffer (20 mM HEPES, 100 mM NaCl (pH 7.2)). Fluorescent gel images (Fl) of the SDS-PAGE show that chemical labeling of S1S2J using **CAM2(OG)**, which was inhibited in the presence of 100 μM of NBQX at the labeling step. CBB indicates Coomassie brilliant blue (CBB) stained gel image. (**b**) The primary sequence of S1S2J and the assignment of each fragment generated by trypsin digestion. The digested peptide fragments are named as T1−T26. (**c**) RP-HPLC traces of trypsin-digested S1S2J labeled with **CAM2(OG)**. The peaks were monitored by fluorescence detection (λ_{ex} = 450 nm, λ_{em} = 550 nm), and assigned by MALDI-TOF MS/MS analyses. (**d**) Representative MALDI-TOF MS/MS analyses of the OG+T6+T7 fragment. The MS/MS data indicates that Lys60 in GluA2(S1S2J) was labeled in this fragment. Labeling sites of other fragments in **c** were determined by similar procedures.

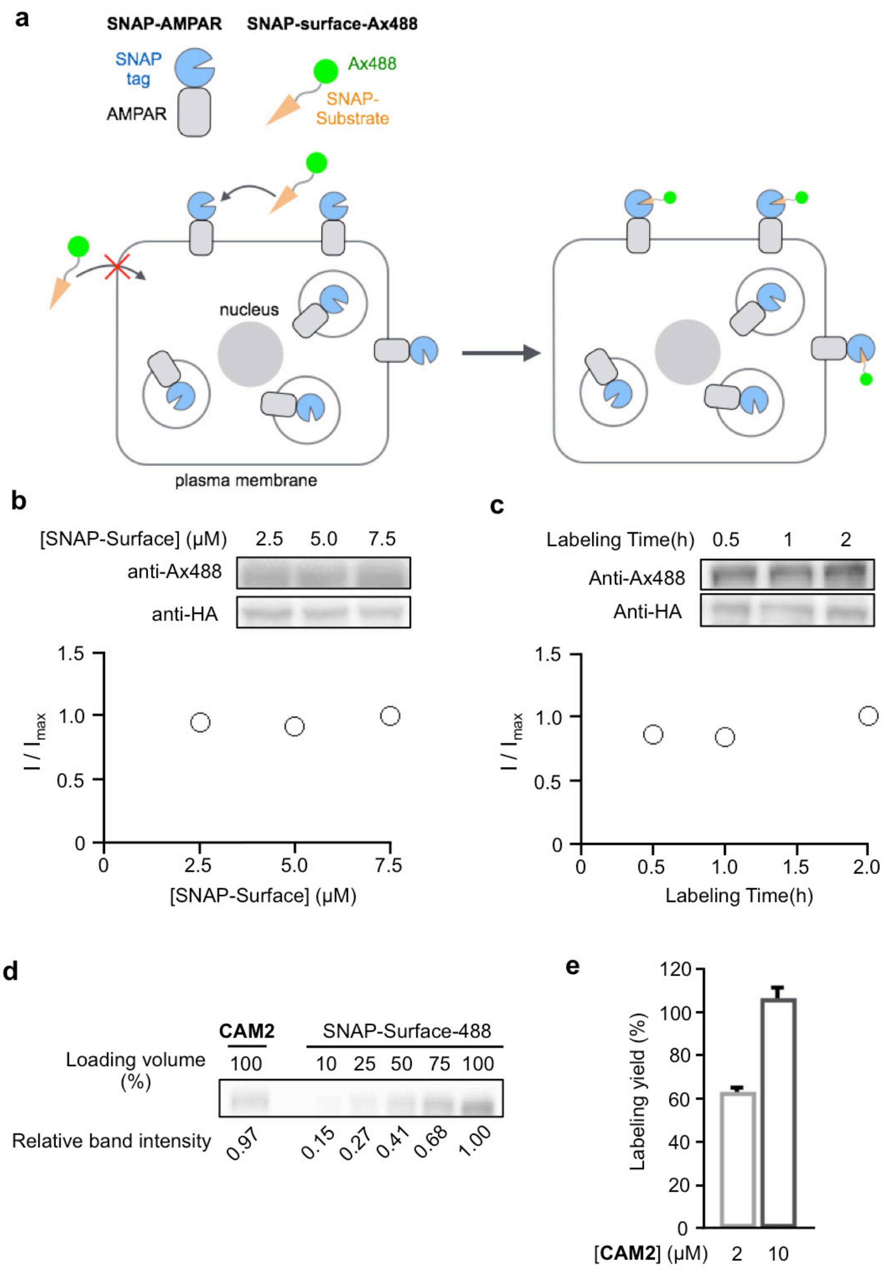

Supplementary Figure 2 | Determination of labeling yield of CAM2 reagents to surface exposed GluA2 by comparing Ax488-conjugated SNAP-AMPARs. (a) Schematic illustration of Alexa488 labeling to SNAP-AMPARs (SNAP-tag was fused to *N*-terminus of GluA2) on live cells. Surface exposed SNAP-AMPARs (GluA2^{flip}(Q)) can be selectively labeled with Ax488 using SNAP-Surface® Alexa Fluor®488 (SNAP-surface-Ax488). (**b**, **c**) Concentration dependency (in **b**) and time dependency (in **c**) of Ax488 labeling using SNAP-surface-Ax488 on live cells. The

SNAP-labeling was conducted for 0.5 h at 17 °C in **b**, and 5.0 μM SNAP-surface-Ax488 was treated at 17 °C in **c**. Ax488 labeling was examined by western blotting using an anti-Ax488 antibody. These results indicate that surface exposed SNAP-AMPARs were fully labeled with Ax488 by incubation with 5 μM SNAP-surface-Ax488 for 0.5 h. According to these results, we concluded that SNAP-AMPARs on the cell surface are completely labeled with 5 μM SNAP-surface-Ax488 for 0.5 h at 17 °C. (**d**, **e**) Determination of labeling yield of **CAM2(Ax488)** to surface exposed GluA2. HEK293T cells transfected with SNAP-AMPARs (SNAP-GluA2) were labeled with 2 or 10 μM of **CAM2(Ax488)** for 4 h at 17 °C, or labeled with 5 μM SNAP-surface-Ax488 for 0.5 h at 17 °C. Band intensity in western blot analysis by **CAM2(Ax488)** to SNAP-AMPARs was compared with that obtained by SNAP-surface-Ax488 to SNAP-AMPARs. In **d**, the representative western blot image is shown. In **e**, labeling yield is indicated. The labeling yield to surface exposed AMPARs on live cells using 2 μM or 10 μM of **CAM2(Ax488)** for 4 h was determined to be 62.0 ± 2.4% (n = 3) and 106 ± 6 % (n = 3), respectively. Data are represented as mean ± SEM.

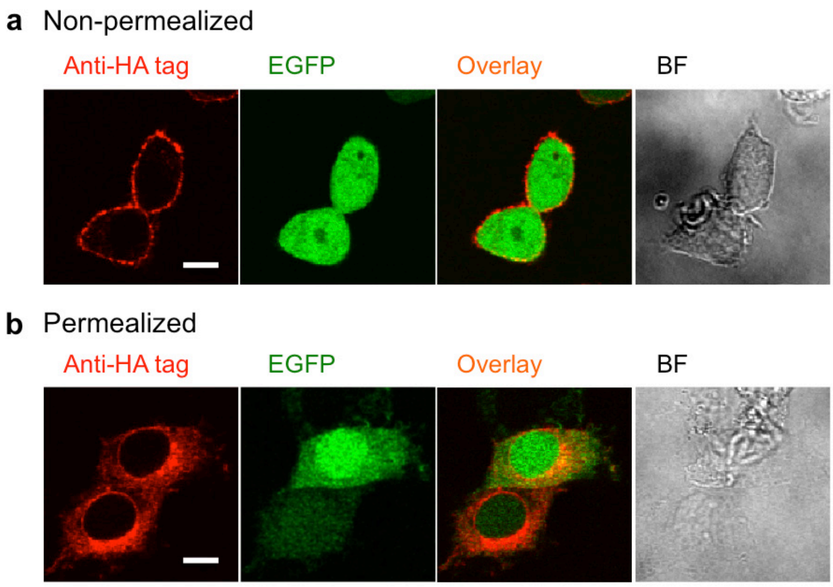

Supplementary Figure 3 | Immunostaining of GluA2 in HEK293T cells. (a) Immunostaining of surface exposed AMPARs. Live HEK293T cells transfected with HA-tagged GluA2^{flip}(Q) were immunostained with a Dylight 550-conjugated anti-HA-tag antibody. Representative confocal images are shown. EGFP was utilized as a transfection marker. Scale bar, 10 μm. (**b**) Immunostaining of whole AMPARs. HEK293T cells transfected with HA-tagged GluA2^{flip}(Q) were fixed and permeabilized with 0.2% triton. Then, the cells were immunostained with a Dylight 550-conjugated anti-HA-tag antibody. Representative confocal images are shown. EGFP was utilized as a transfection marker. Scale bar, 10 μm.

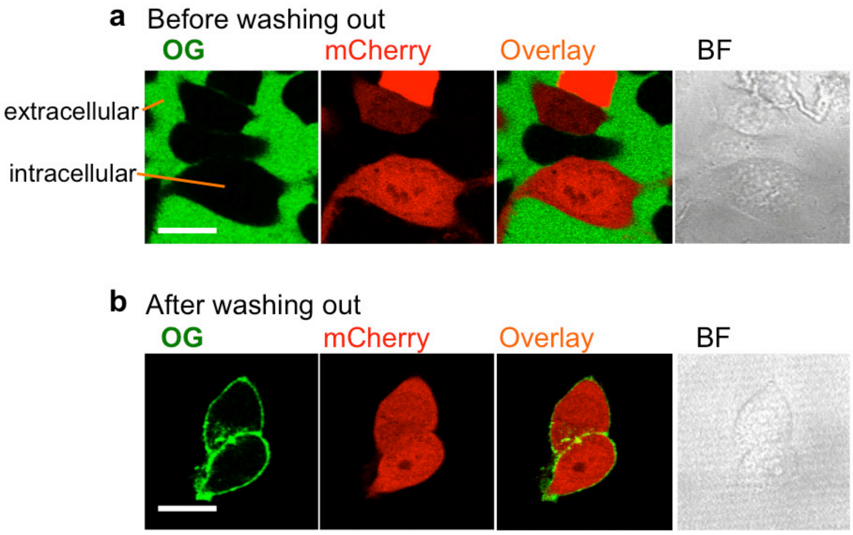

Supplementary Figure 4 | CAM2(OG) dose not have permeability into live cells. (**a**, **b**) Representative confocal images of HEK293T cells transfected with GluA2^{flop}(R). The cells were incubated with 2 μM **CAM2(OG)** for 4 h at 17 ℃. In **a**, the image before washing out the medium is shown. In **b**, the image after washing out the medium three times is shown. mCherry was utilized as a transfection marker. Scale bars, 20 μm.

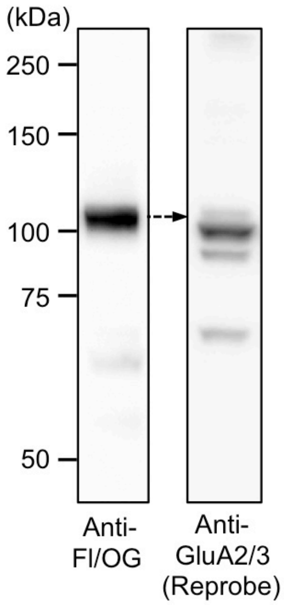

Supplementary Figure 5 | Reciprocal immunoblot analyses of labeled AMPAR using anti-Fl/OG and anti-GluA2 antibodies. HEK293T cells transfected with GluA2 ^{flop}(R) was labeled with 2 μM of **CAM2(OG)** in serum free DMEM. After analyses of the cell lysate by western blot analysis using anti-Fl/OG antibody, the membrane was stripped and reprobed with anti-GluA2/3 antibody. These data indicate that the single band in the blot using anti-Fl/OG antibody merged well with the highest molecular weight band visualized with anti-GluA2/3 antibody.

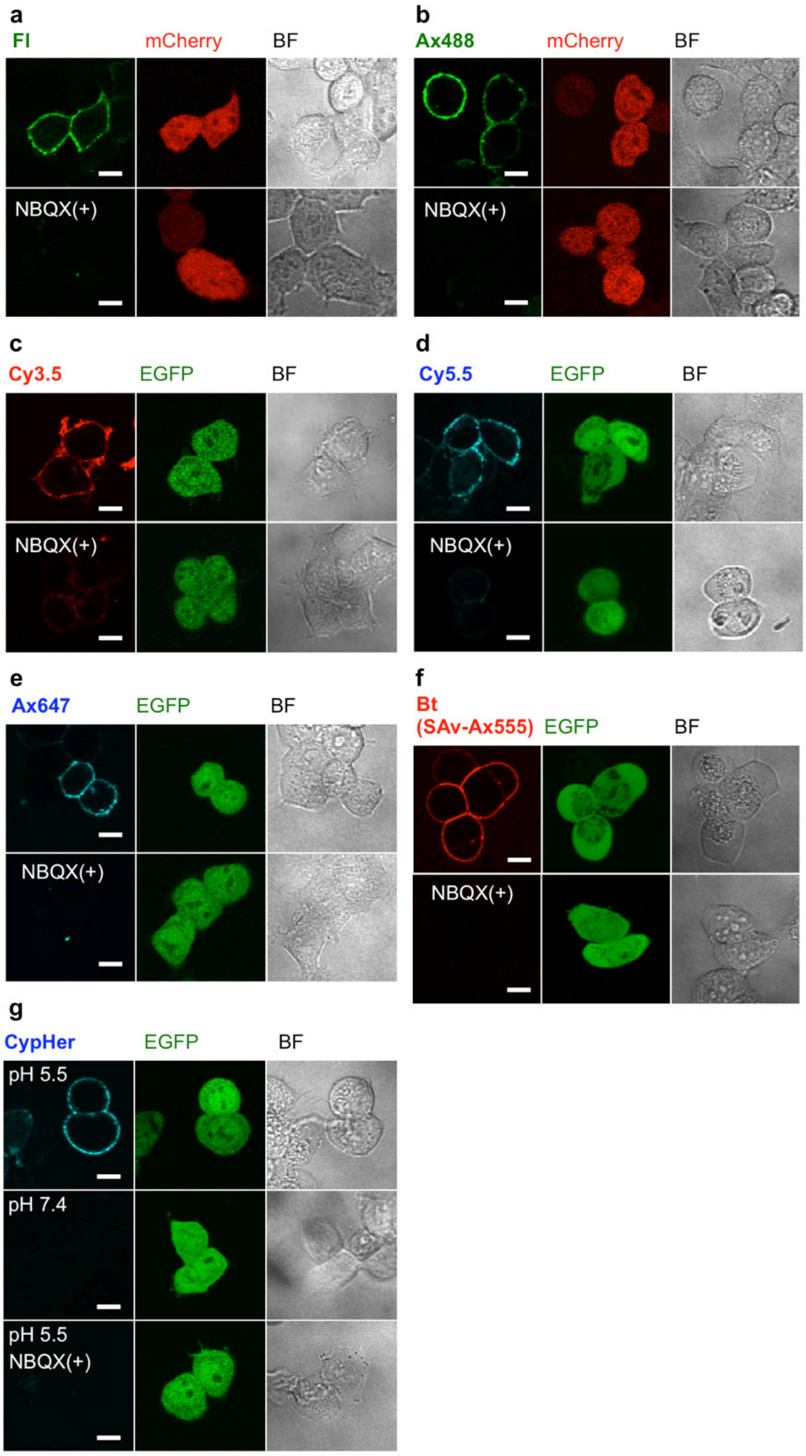

Supplementary Figure 6 | Chemical labeling of AMPARs on live HEK293T cells by various chemical probes. HEK293T cells transfected with GluA2^{flip}(Q) were labeled with 3 μM of **CAM2(Fl)** (in **a**), **CAM2(Ax488)** (in **b**), **CAM2(Cy3.5)** (in **c**), **CAM2(Cy5.5)** (in **d**), **CAM2(Ax647)** (in **e**), **CAM2(Bt)** (in **f**), or **CAM2(CypHer)** (in **g**) in the presence or absence of 50 μM NBQX in serum free DMEM.

Representative confocal images are shown. In **f**, the cells were stained with a streptavidin-Ax555 conjugate (SAv-Ax555) after labeling with **CAM2(Bt)**. In **g**, the cells labeled with **CAM2(CypHer)** in the absence of NBQX were imaged in MES buffered saline at pH 5.5 (20 mM MES, 107 mM NaCl, 6 mM KCl, 2 mM $CaCl_2$, and 1.2 mM $MgSO_4$) (in top) or HBS at pH 7.4 (in middle). This indicates that labeled **CypHer** shows high fluorescence under acidic conditions. EGFP or mCherry was utilized as a transfection marker. Scale bars, 10 μm.

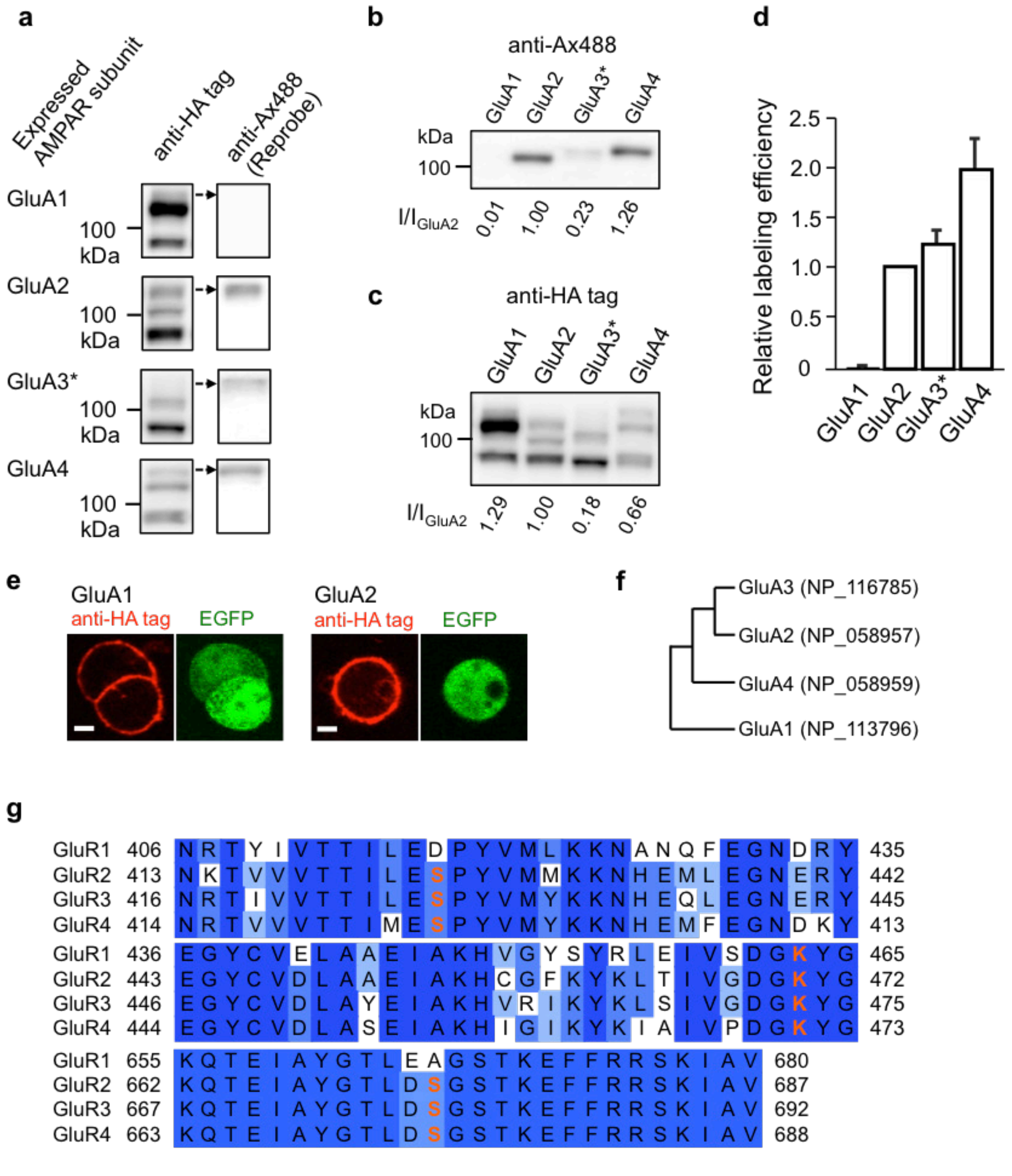

Supplementary Figure 7 | AMPAR subunit selectivity of chemical labeling by CAM2 reagents. (**a**) Reciprocal immunoblot analyses of labeled AMPARs using anti-HA tag and anti-Ax488 antibodies. HEK293T cells transfected with each HA tagged AMPAR subunit (GluA1^{flip}(Q), GluA2^{flip}(Q), GluA3^{flip}(Q)* or GluA4^{flip}(Q)) were treated with 2 μM of **CAM2(Ax488)** in serum free DMEM. After analyses of each cell lysate by western blot analysis using anti-HA tag antibody, the membrane was stripped and reprobed with anti-Ax488 antibody. These data indicate that the single labeling band observed in the blots analyzing GluA2–4 expressing cell lysates by using Ax488 antibody merged well with the highest molecular weight band visualized with anti-HA tag antibody. GluA3^{flip}(Q)* indicates GluA3 (Y454A/R461G)

mutant in which surface expression of GluA3 homomer is enhanced[1]. (**b**) Comparison of the labeling efficiency among the different subunits (GluA1–4). The representative image of western blot using anti-Ax488 antibody is shown. The relative labeling band intensity to GluA2 (I/I_{GluA2}) is also indicated. (**c**) Comparison of the surface expression levels among the different subunits. The representative image of western blot using anti-HA tag antibody is shown. The relative intensity (I/I_{GluA2}) of the highest molecular weight bands to that of GluA2 is also indicated. (**d**) Comparison of the labeling efficiency among the different subunits. The labeling band intensity of each subunit in **b** was normalized with surface expression levels in **c** (n = 3). (**e**) Immunostaining of surface exposed AMPARs. Live HEK293T cells transfected with HA-tagged GluA1^{flip}(Q) or GluA2^{flip}(Q) were immunostained with a Dylight 550-conjugated anti-HA-tag antibody. Representative confocal images are shown. EGFP was utilized as a transfection marker. The data indicates that GluA1 as well as GluA2 are properly expressed on cell surface. Scale bars, 5 μm. (**f**) The phylogenetic tree of AMPAR subunits constructed using a sequence alignment program (ClustalW). This indicates that GluA1 shows low homology among AMPAR subunits. (**g**) Multiple sequence alignment for AMPAR subunits in rats. Labeling sites with **CAM2** are shown in orange (see also **Fig. 2b** and **Supplementary Fig. 1**).

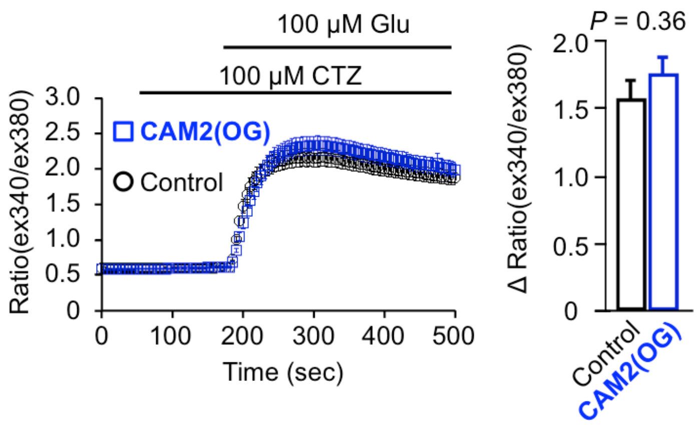

Supplementary Figure 8 | Ca^{2+} responses of AMPARs are not affected by the chemical labeling procedure in HEK293T cells. HEK293T cells transfected with Ca^{2+}-permeable GluA2 ($GluA2^{flip}$(Q)) was labeled with 3 μM of **CAM2(OG)** in serum free DMEM, and subjected to intracellular Ca^{2+} concentration ($[Ca^{2+}]_i$) measurements by using a Ca^{2+} indicator, Fura-2. The cells not treated with **CAM2(OG)** was utilized as the control. Cyclothiazide (CTZ) and glutamate (Glu) were applied during periods indicated by bars, and $[Ca^{2+}]_i$ changes (340/380 nm excitation fluorescence ratio; ratio(ex340/ex380)) evoked by 100 μM glutamate (Glu) were measured. Left, averaged time courses. Right, maximal $[Ca^{2+}]_i$ rises (Δratio(ex340/ex380)) (n = 87–96). Data points are mean ± SEM. Student's *t*-test indicates that these are not significantly different.

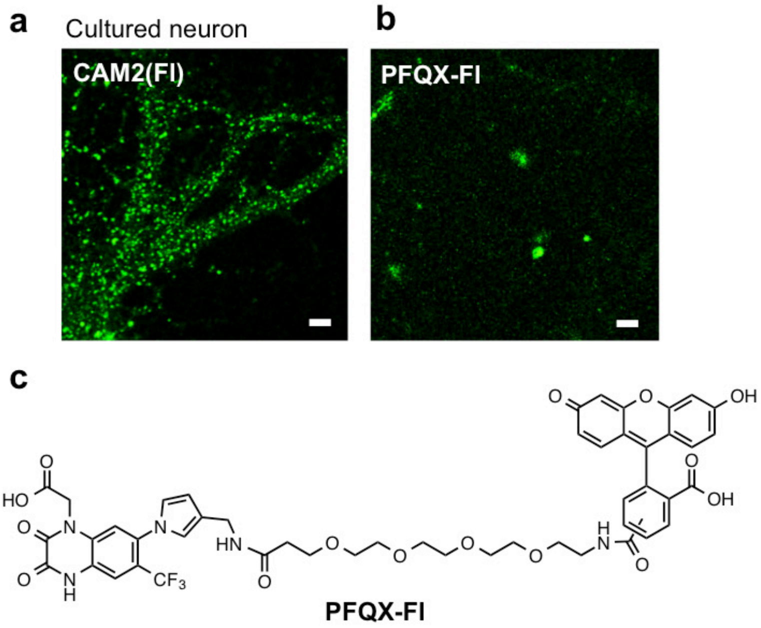

Supplementary Figure 9 | Live imaging of endogenous AMPARs in cultured hippocampal neurons. (**a**) Confocal imaging of cultured hippocampal neurons labeled with **CAM2(Fl)**. Hippocampal neurons were treated with 1 μM of **CAM2(Fl)**, and washed out three times with ACSF. (**b**) Confocal imaging of cultured hippocampal neurons treated with **PFQX-Fl**, an analogue to **CAM2(Fl)** lacking the reactive moiety for covalent labeling. Hippocampal neurons were treated with 1 μM of **PFQX-Fl** and washed out three times with ACSF. The data indicates that **PFQX-Fl** can be washed out under the live imaging condition. Scale bars, 10 μm. (**c**) Chemical structure of **PFQX-Fl**.

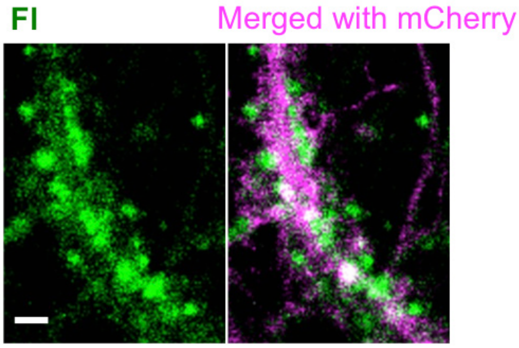

Supplementary Figure 10 | Confocal live imaging of labeled AMPARs with CAM2(Fl) in cultured neurons. The cultured hippocampal neurons were transfected with mCherry to visualize the dendrite. Hippocampal neurons were treated with 1 μM of **CAM2(Fl)**, and washed out three times with ACSF. Scale bar, 2 μm.

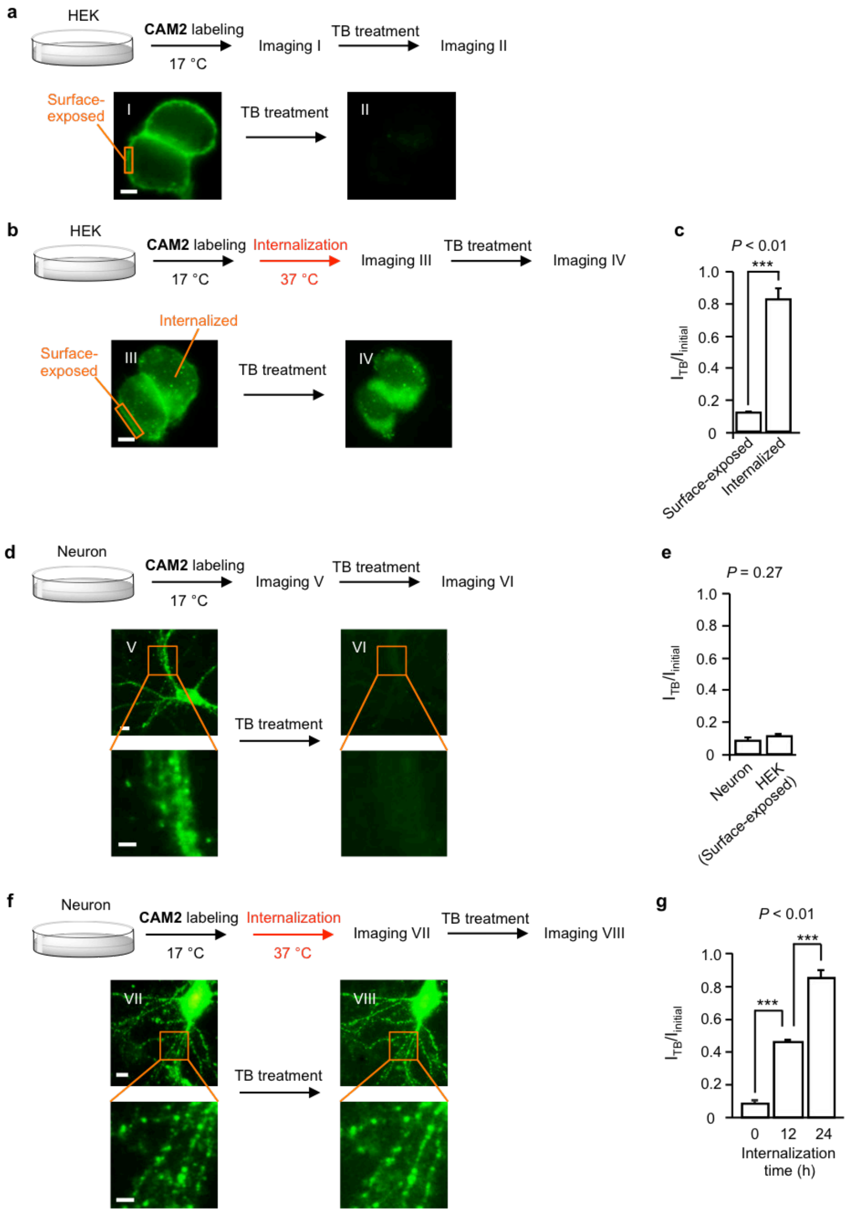

Supplementary Figure 11 | CAM2 reagent mainly labels surface-exposed AMPARs in HEK293T cells or cultured hippocampal neurons. (**a**) HEK293T cells transiently expressing GluA2^{flip}(Q) were treated with 2 μM of **CAM2(Ax488)** for 4 h at 17 °C. Labeled GluA2 were predominantly observed from cell surface by epifluorescent microscopy (left). Treatment with 0.4% trypan blue (TB) quenched most fluorescent signals (right). Scale bar, 5 μm. (**b**) After chemical labeling by 2 μM of **CAM2(Ax488),** the cells were incubated for 2 h at 37 °C to promote internalization

of labeled GluA2. Labeled GluA2 were observed mainly from intracellular regions and partly from cell surface (left). TB treatment failed to quench intracellular fluorescence, although fluorescence from cell-surface was quenched in this condition. (right). Scale bar, 5 μm. (**c**) Comparison of quenching ratio ($I_{TB}/I_{initial}$) obtained from surface-exposed GluA2 (n = 6) and internalized GluA2 (n = 7) as shown in **b**. This indicates that TB treatment selectively quenches the fluorescence of surface-exposed labeled GluA2. (**d**) Cultured hippocampal neurons labeled by 1 μM of **CAM2(Ax488)** were subjected to TB treatment. Orange square ROIs indicated in upper panels are expanded in lower panels. Scale bars, 10 μm in upper panels and 5 μm in lower panels. (**e**) Comparison of $I_{TB}/I_{initial}$ obtained from hippocampal neurons (in **d**, n = 12) and HEK293T cells (in **b**, n = 6). Student's t-test indicates that significant differences were not observed, which indicates that surface-exposed AMPARs were predominantly visualized by the **CAM2** reagent in cultured hippocampal neurons. Data points mean ± SEM. (**f**) After chemical labeling by 1 μM of **CAM2(Ax488)**, the neurons were incubated for 24 h at 37 °C to promote internalization of labeled AMPARs and subjected to TB treatment. Orange square ROIs indicated in upper panels are expanded in lower panels. Scale bars, 10 μm in upper panels and 5 μm in lower panels. (**g**) Comparison of $I_{TB}/I_{initial}$ obtained from hippocampal neurons after incubation at 37 °C for 0 h (in **d**, n = 12), 12 h (the image not shown, n = 11) or 24 h (in **f**, n = 12). This data also supports that surface-exposed AMPARs are predominantly visualized immediately after **CAM2** labeling.

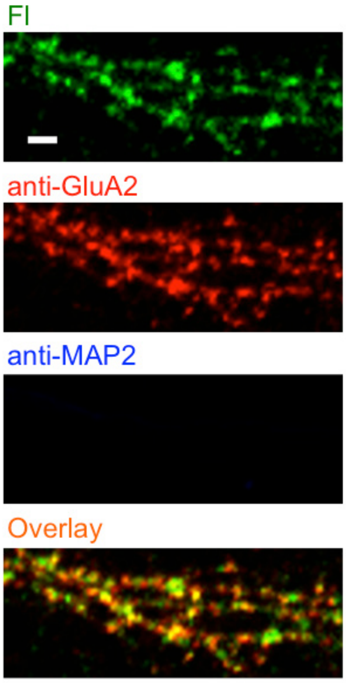

Supplementary Figure 12 | Surface immunofluorescence staining of cultured neurons after chemical labeling with CAM2. Cultured hippocampal neurons labeled with 1 μM **CAM2(Fl)** were fixed with paraformaldehyde and immunostained using anti-GluA2 and anti-MAP2 without triton permeabilization. Representative confocal images are shown. Scale bar, 2 μm.

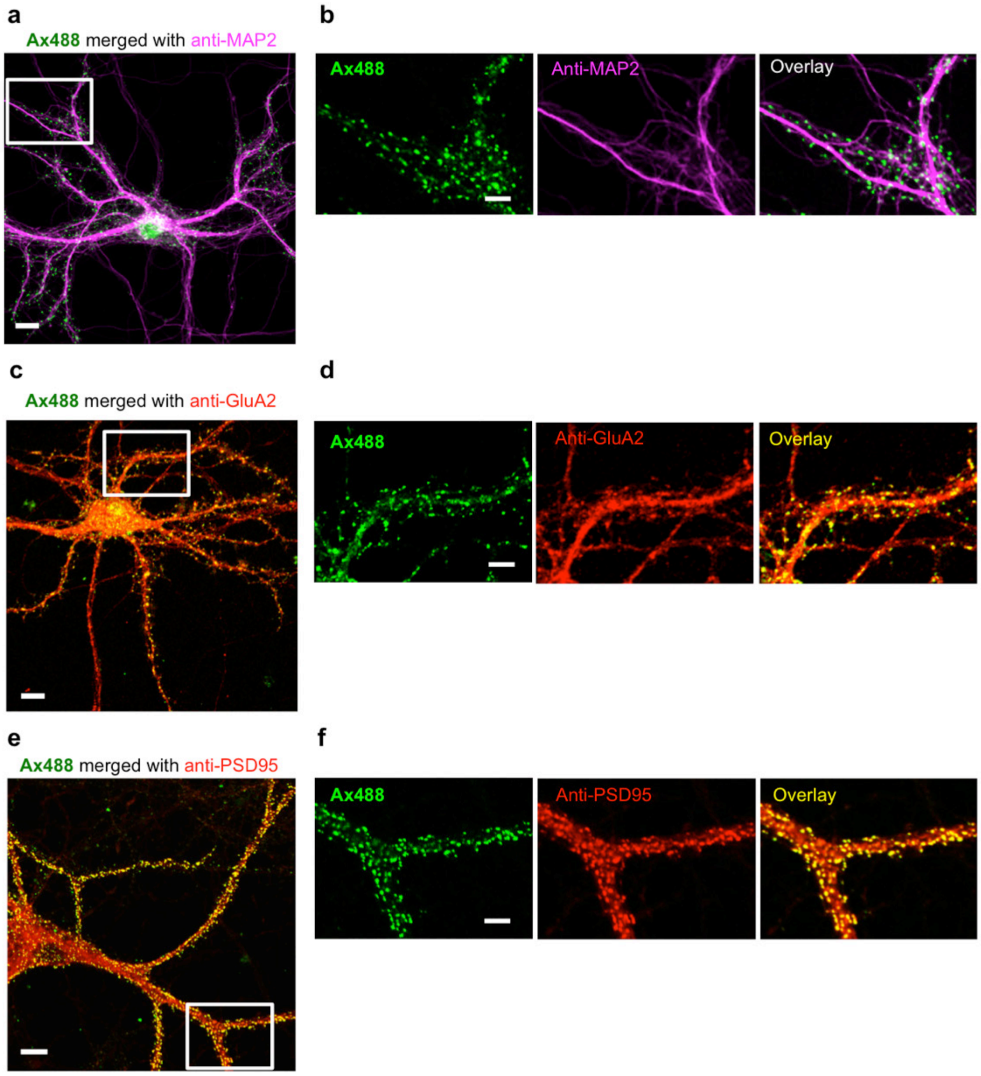

Supplementary Figure 13 | Confocal imaging of cultured neurons after labeling using CAM2(Ax488) reagents. Cultured hippocampal neurons labeled by 1 μM of **CAM2(Ax488)** was fixed, permeabilized and immunostained with an anti-MAP2 (in **a** and **b**), anti-GluA2 (in **c** and **d**) or anti-PSD95 antibody (in **e** and **f**). White square ROIs indicated in **a**, **c**, and **e** are expanded in **b**, **d**, and **f**, respectively. Scale bars, 10 μm (**a**, **c**, and **e**) and 3 μm (**b**, **d**, and **f**).

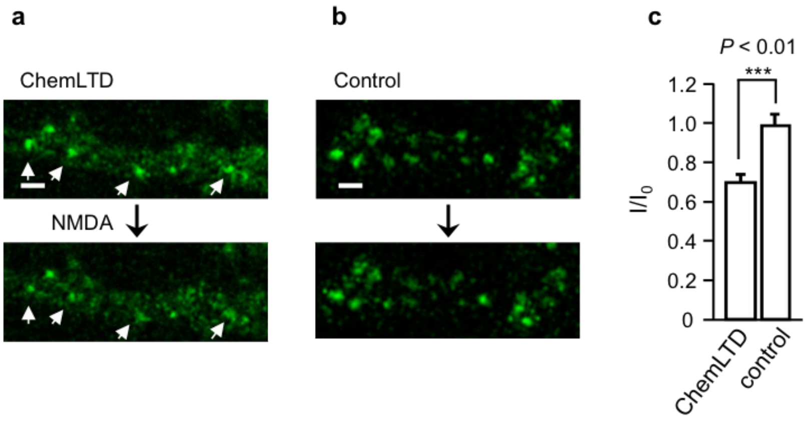

Supplementary Figure 14 | Removal of AMPARs from spine by chemLTD induction. Hippocampal neurons after labeling using **CAM2(Fl)** were treated with 50 μM of NMDA for 10 min. (**a**) Representative confocal images of labeled hippocampal neurons before and after NMDA stimulation. Scale bars, 2 μm. (**b**) Representative confocal images of the control experiment without NMDA stimulation. Scale bars, 2 μm. (**c**) Comparison of fluorescence ratio (I/I_0) with or without chemLTD induction (n = 10). Data are represented as mean ± SEM.

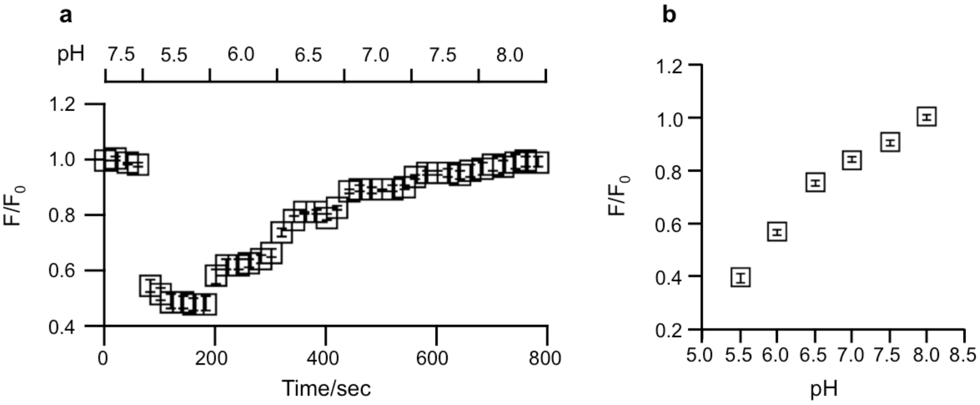

Supplementary Figure 15 | pH sensitivity of Fl-AMPARs on live HEK293T cells. HEK293T cells transfected with GluA2^{flop}(R) were labeled with 2 μM of **CAM2(Fl)**. Extracellular pH was stepwise changed from 7.5 to 5.5, to 6.0, to 6.5, to 7.0, to 7.5 and finally to 8.0. The cellular fluorescence at different pH was monitored (n = 41). (**a**) Representative fluorescent trace recorded from the labeled cells. Initial fluorescence in this measurement at pH 7.5 is defined as F_0. (**b**) Fluorescent ratio (F/F_0) at different pH. Data points mean ± SEM.

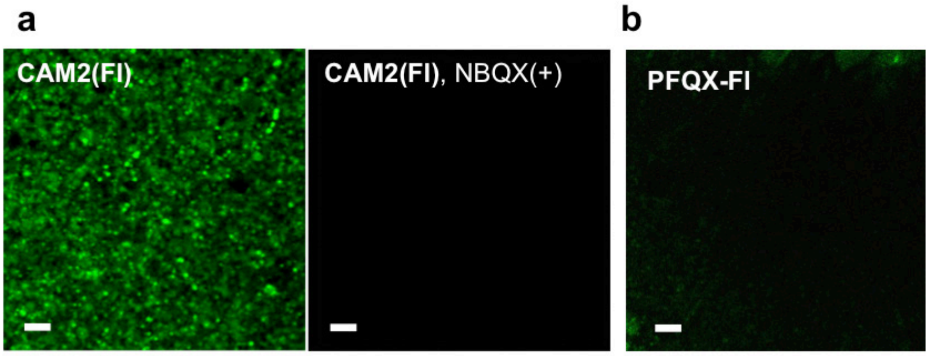

Supplementary Figure 16 | Live imaging of endogenous AMPARs in hippocampal slices. (**a**) Chemical labeling of endogenous AMPARs with **CAM2(Fl)** or suppression of chemical labeling in the presence of competitive inhibitor in hippocampal slices. Acutely prepared hippocampal slices were treated with 1 μM of **CAM2(Fl)** in the absence (in left) or presence (in right) of 10 μM NBQX in ACSF, and washed out three times with ACSF. If the washing procedure is insufficient, fluorescence from excess **CAM2(Fl)** may be included. Scale bars, 5 μm. (**b**) Confocal live imaging of hippocampal slices treated with **PFQX-Fl**, an analogue to **CAM2(Fl)** lacking the reactive moiety for covalent labeling (see **Supplementary Fig. 9**). Hippocampal slices were treated with 1 μM of **PFQX-Fl** and washed out three times with ACSF. Scale bar, 10 μm. The data indicates that **PFQX-Fl** lacking the reactive moiety can be washed out under the live imaging condition.

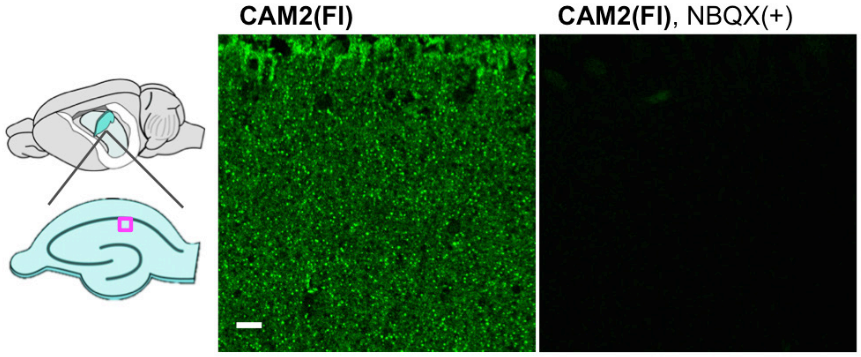

Supplementary Figure 17 | Confocal images of hippocampal brain slices labeled with CAM2(Fl). Hippocampal slices treated with 1 μM of **CAM2(Fl)** in the absence or presence of 10 μM NBQX in ACSF were fixed with paraformaldehyde. Single plane confocal images of labeled slices are shown. In the left, imaged region is shown as a magenta square. Scale bar, 10 μm.

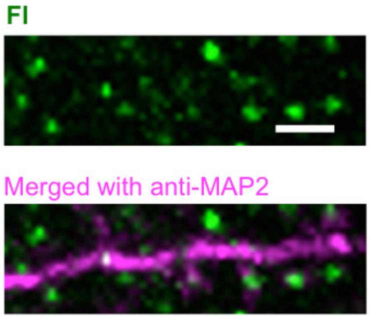

Supplementary Figure 18 | Composite Z-stack imaging of endogenous AMPARs in hippocampal slices immunostained with an anti-MAP2 antibody. Hippocampal slices labeled with 1 μM **CAM2(Fl)** were fixed, permeabilized and immunostained with an anti-MAP2 antibody. Scale bar, 2 μm.

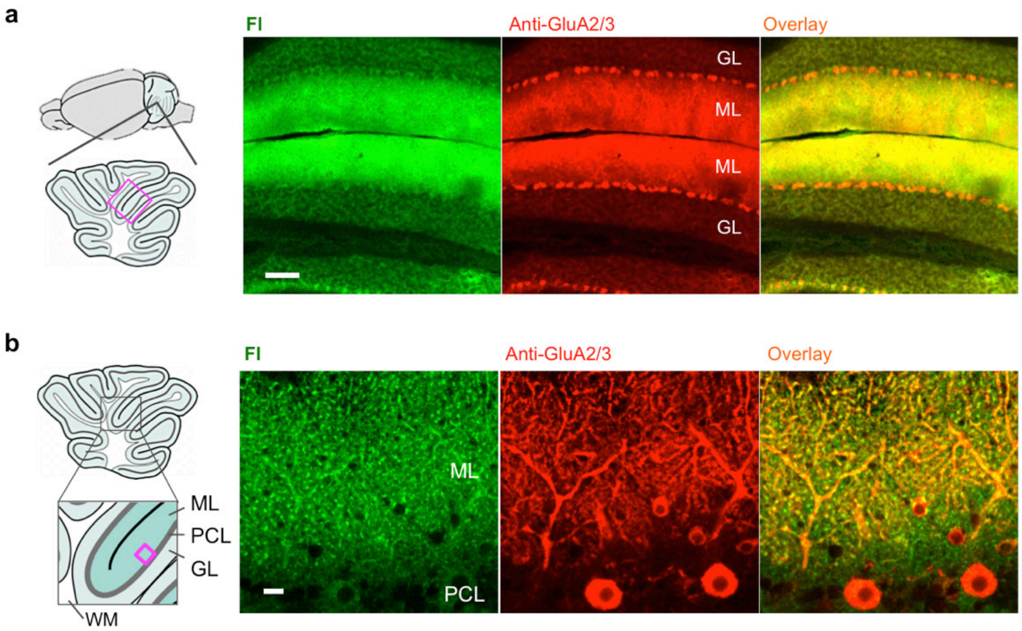

Supplementary Figure 19 | Immunostaining of labeled cerebellar slices with CAM2(Fl). Cerebellar slices treated with 1 μM of **CAM2(Fl)** in ACSF was fixed, permeabilized, and immunostained with anti-GluA2/3 antibody. (**a**, **b**) Single plane confocal images of labeled slices immunostained with anti-GluA2/3 antibody. In the left, imaged region is shown as a magenta square. ML, molecular layer; PCL, Purkinje cell layer; GL, granular layer. In **a**, scale bar, 100 μm. In **b**, magnified images of molecular layers are shown. Scale bar, 10 μm.

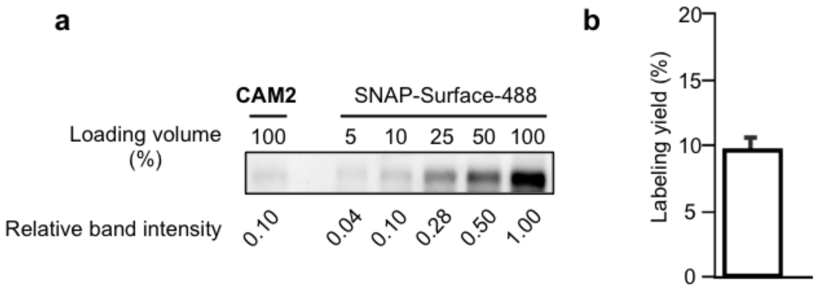

Supplementary Figure 20 | Determination of labeling yield of CAM2 reagents to surface-exposed GluA2 under live imaging condition in cultured neurons. HEK293T cells transfected with SNAP-AMPARs (SNAP-GluA2^{flip}(Q)) were labeled with 1 μM of **CAM2(Ax488)** for 1 h at 17 °C, or labeled with 5 μM of SNAP-surface-Ax488 for 0.5 h at 17 °C. Band intensity in western blot analysis by **CAM2(Ax488)** to SNAP-AMPARs was compared with that obtained by SNAP-surface-Ax488 to SNAP-AMPARs (for details, see **Supplementary Fig. 2**). (**a**) The representative western blot image. (**b**) Calculated labeling yield. The labeling yield to surface exposed AMPARs under live imaging condition in cultured neurons was determined to be 9.6 ± 0.9 % (n = 3). Data are represented as mean ± SEM.

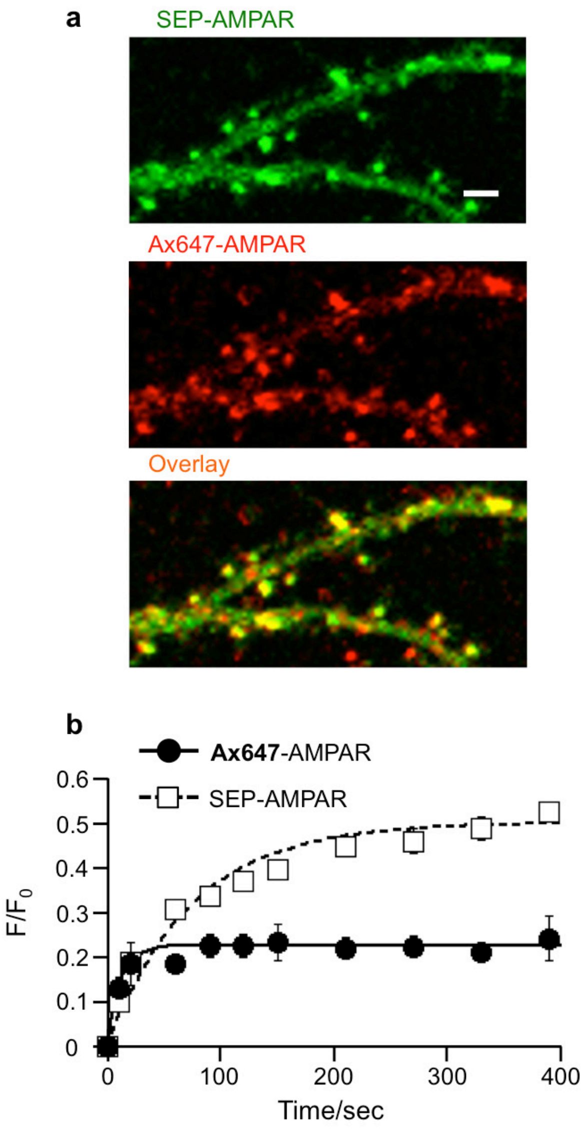

Supplementary Figure 21 | Dual-color FRAP analyses of Ax647-AMPARs and SEP-AMPARs in the same spines. (**a**) Confocal imaging of cultured neurons exogenously expressing SEP-AMPARs after labeling using **CAM2(Ax647)**. Hippocampal neurons transfected with SEP-AMPARs were treated with 1 µM of **CAM2(Ax647)** in ACSF. Scale bar, 2 µm. (**b**) Averaged FRAP curves for **Ax647**-AMPARs and SEP-AMPARs (n = 10). Recovery ratios for **Ax647**-AMPARs and SEP-AMPARs are 22.7 ± 0.7% and 50.2 ± 2.0%, respectively. Data are represented as mean ± SEM.

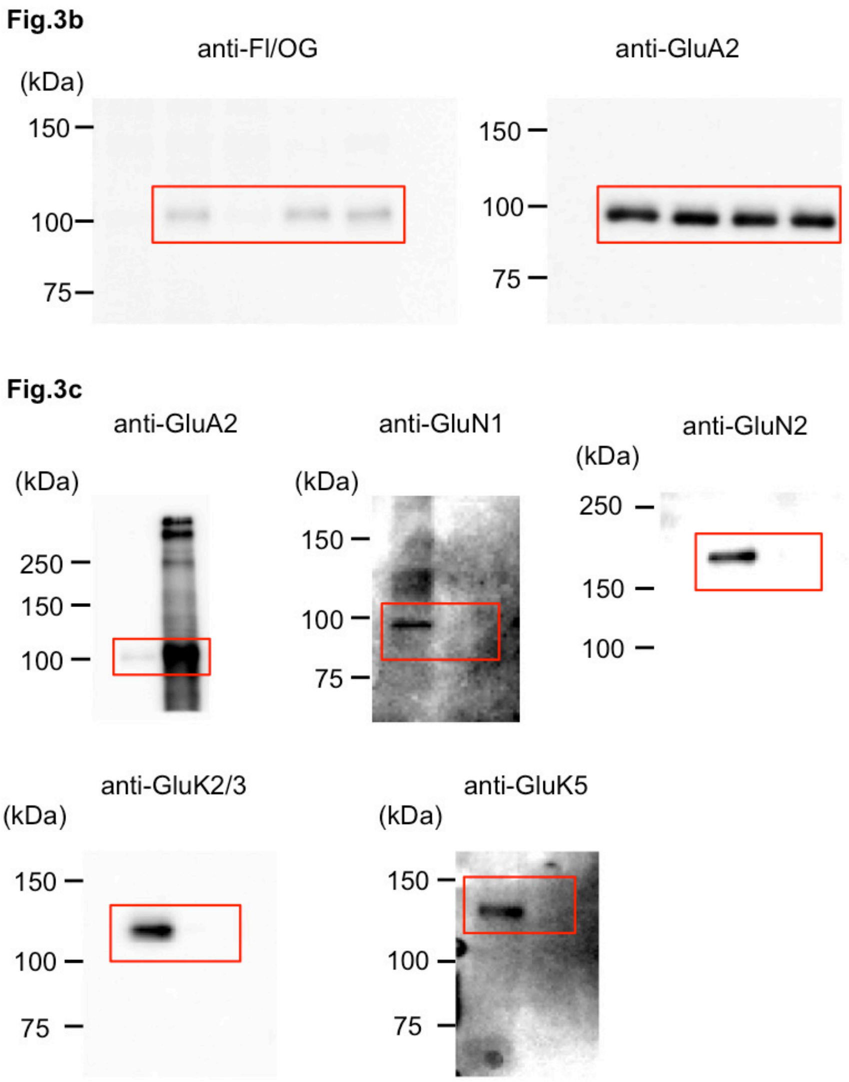

Supplementary Figure 22 | Uncropped scans of the most important western blots.

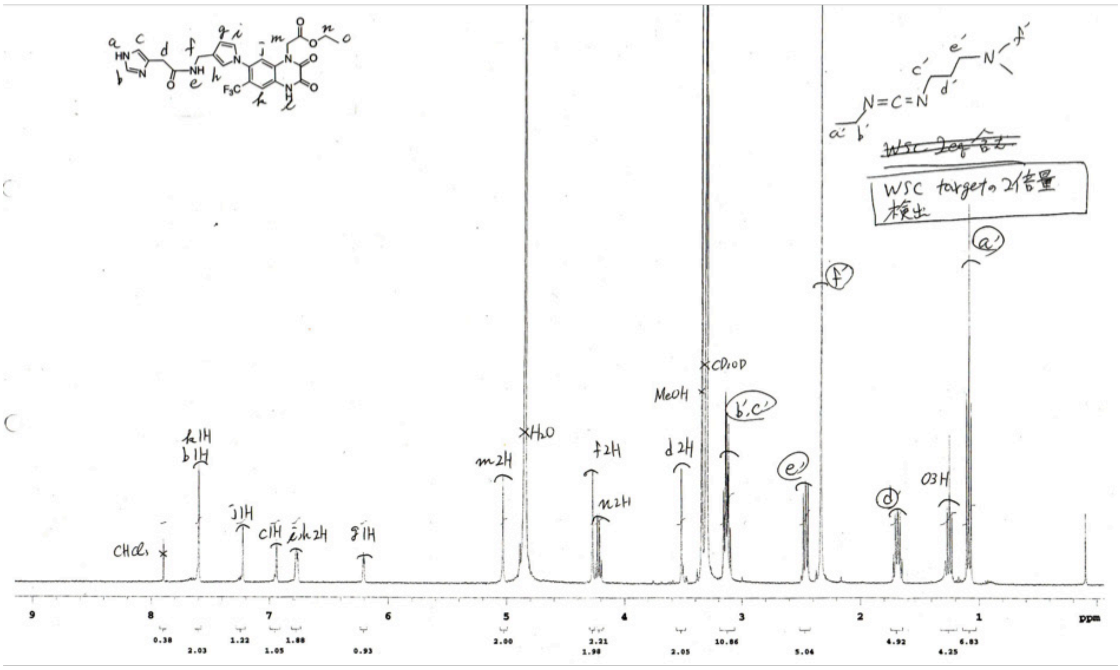

Supplementary Figure 23 | ^{1}H-NMR analysis of compound 7.

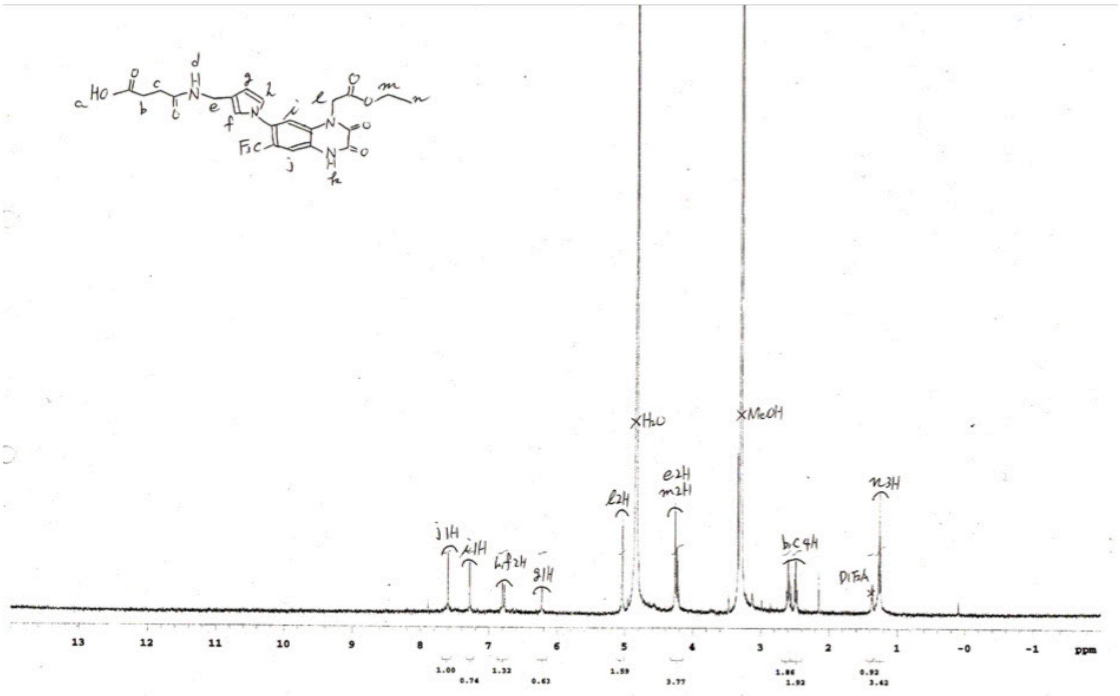

Supplementary Figure 24 | ^{1}H-NMR analysis of compound 5.

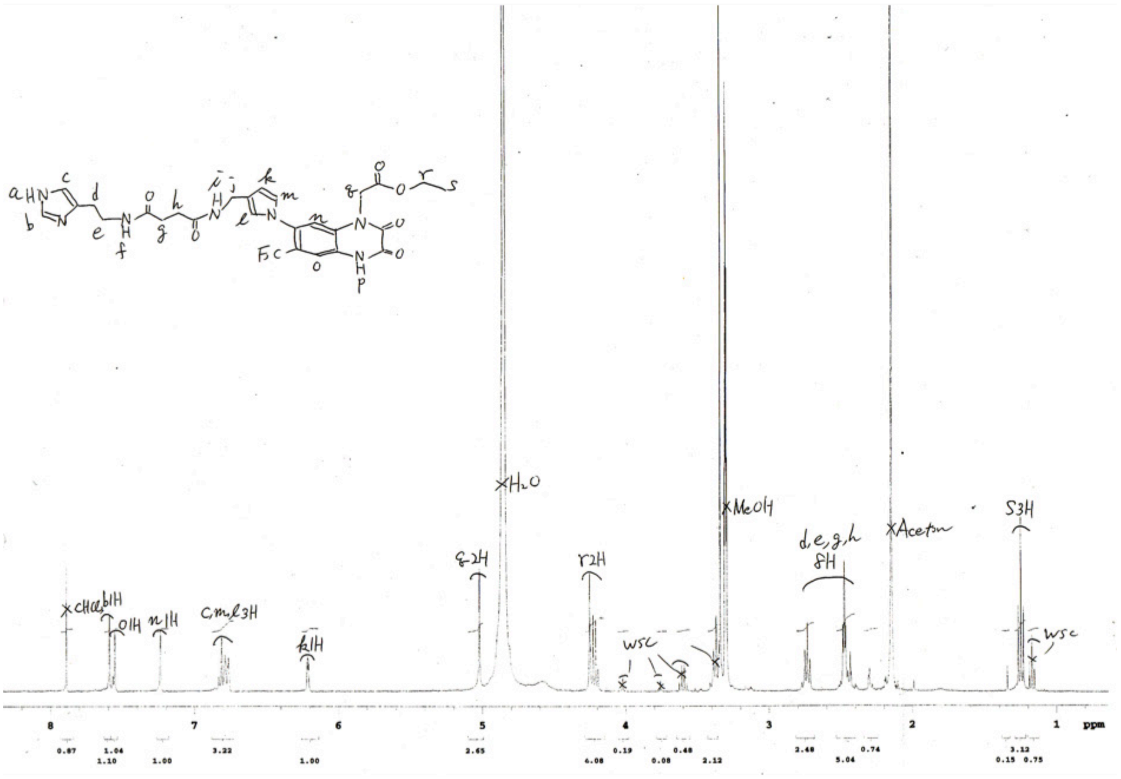

Supplementary Figure 25 | ^{1}H-NMR analysis of compound 8.

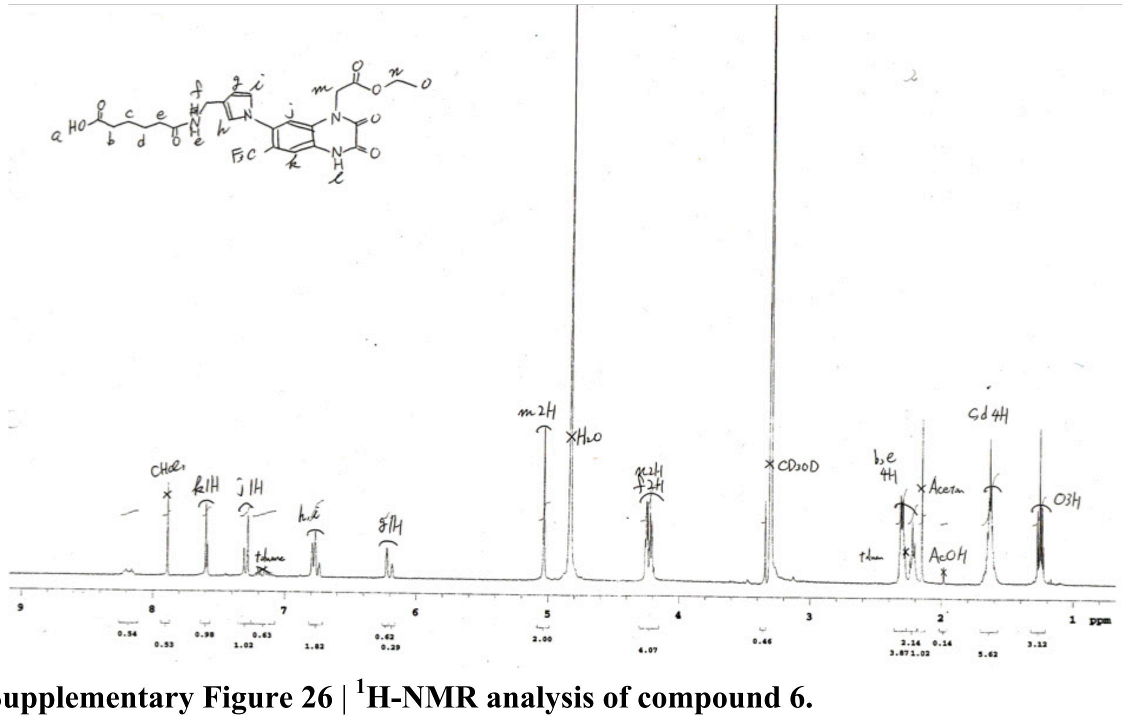

Supplementary Figure 26 | ^{1}H-NMR analysis of compound 6.

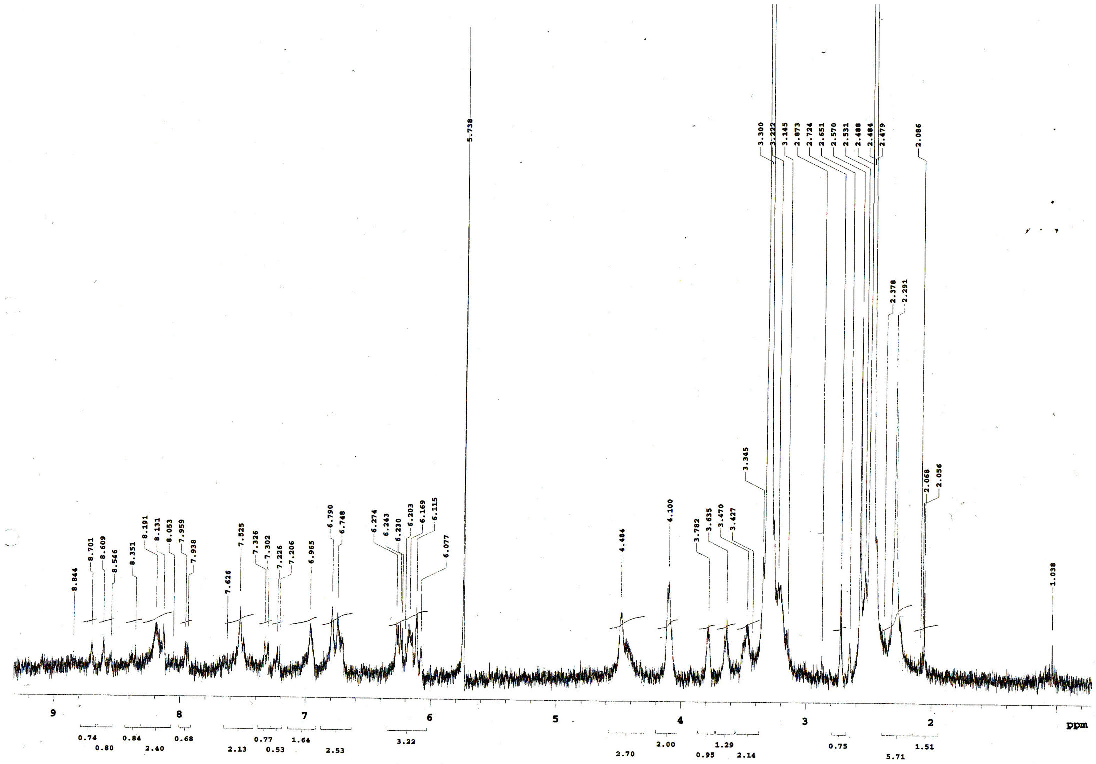

Supplementary Figure 27 | ^{1}H-NMR analysis of CAM2(OG).

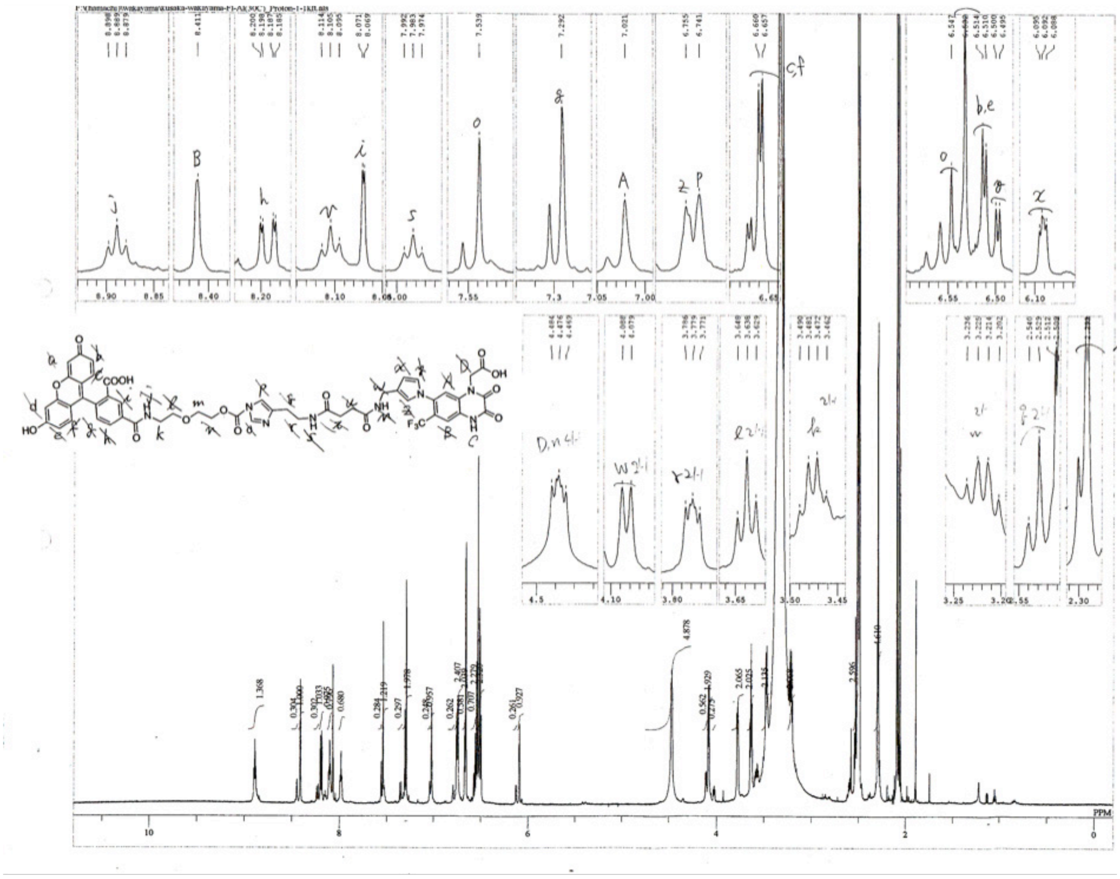

Supplementary Figure 28 | ^{1}H-NMR analysis of CAM2(Fl).

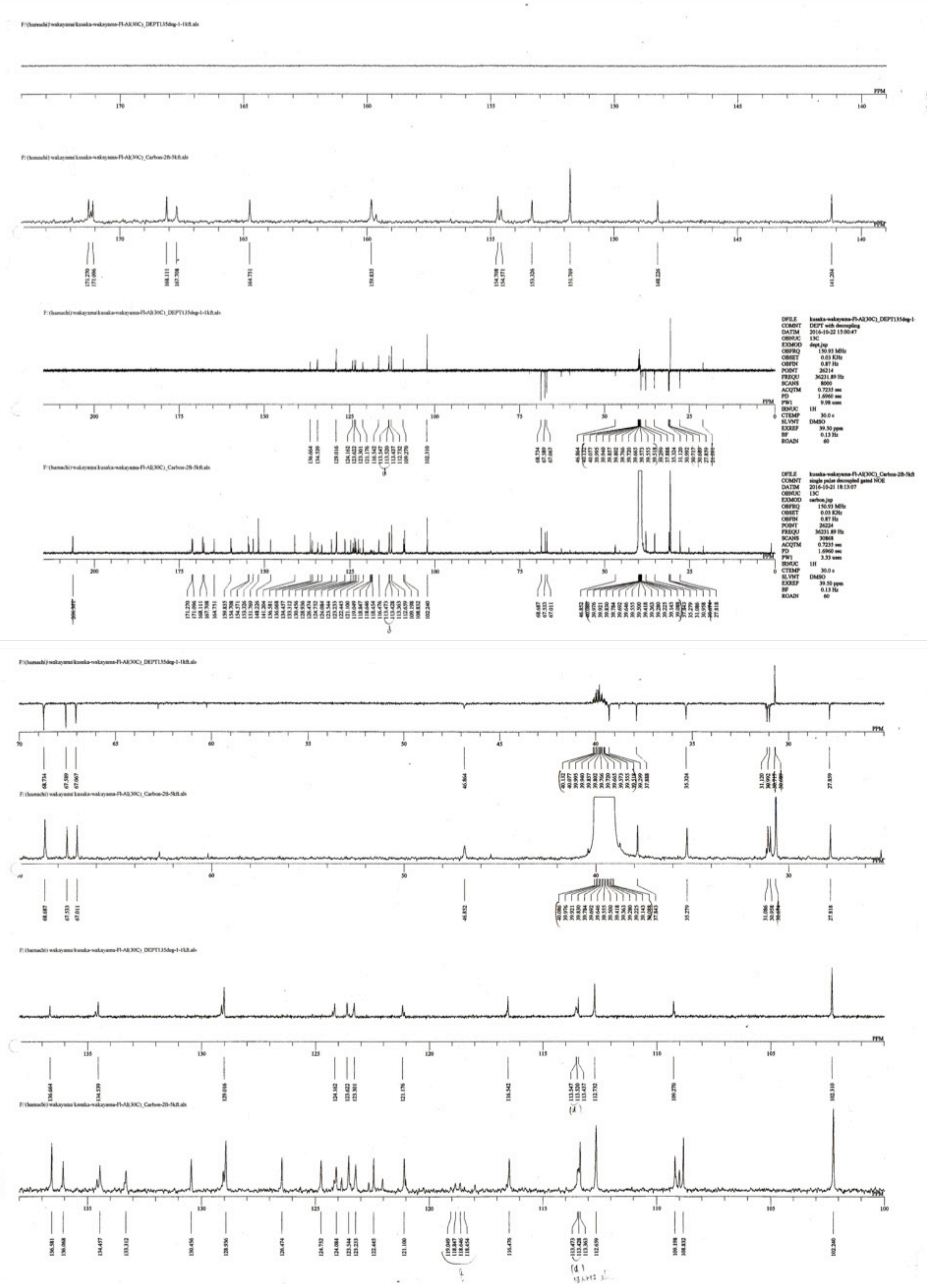

Supplementary Figure 29 | ^{13}C-NMR analysis of CAM2(Fl).

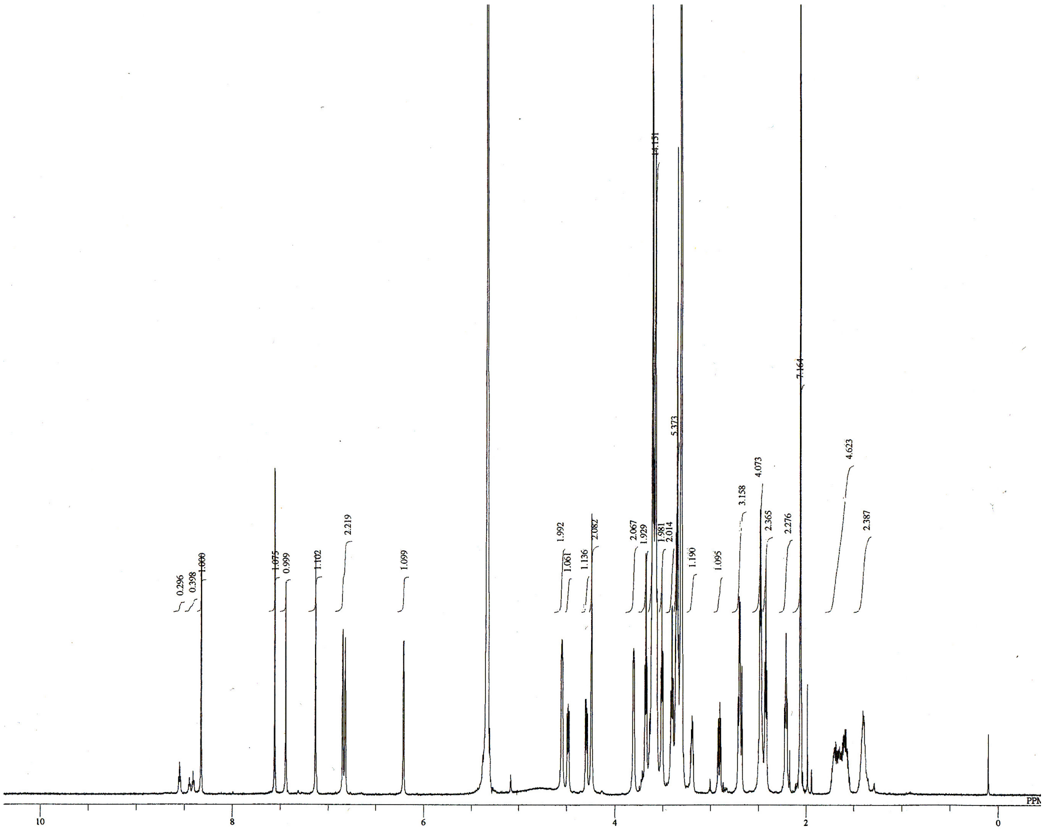

Supplementary Figure 30 | ^{1}H-NMR analysis of CAM2(Bt).

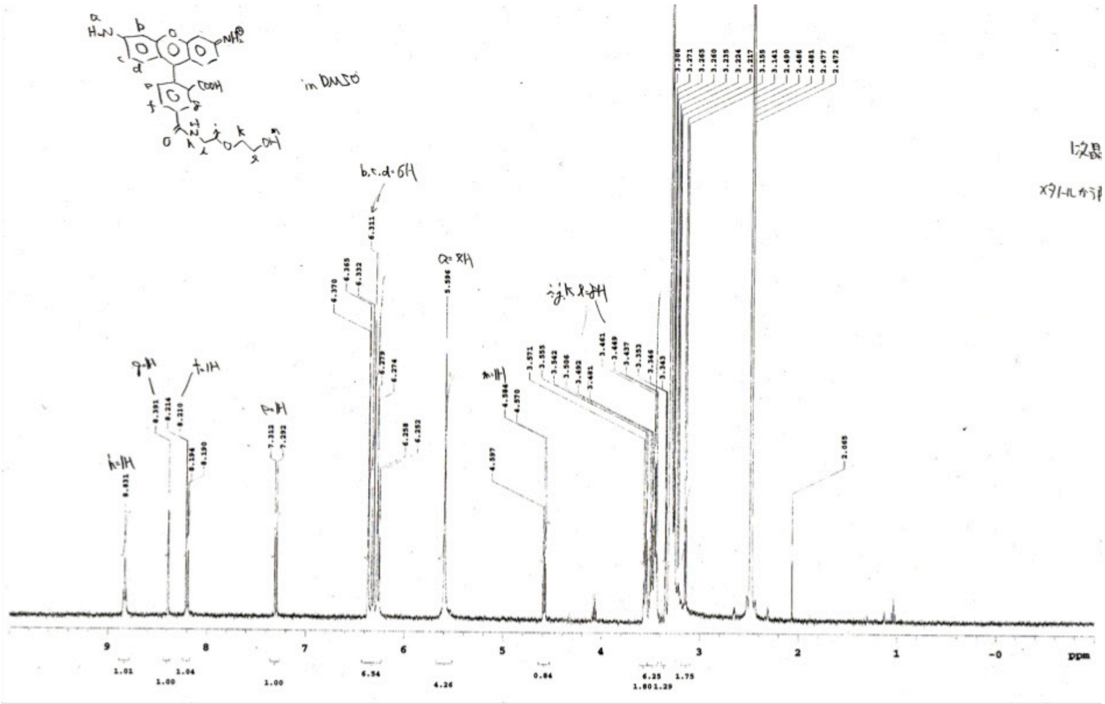

Supplementary Figure 31 | ^{1}H-NMR analysis of Compound 17.

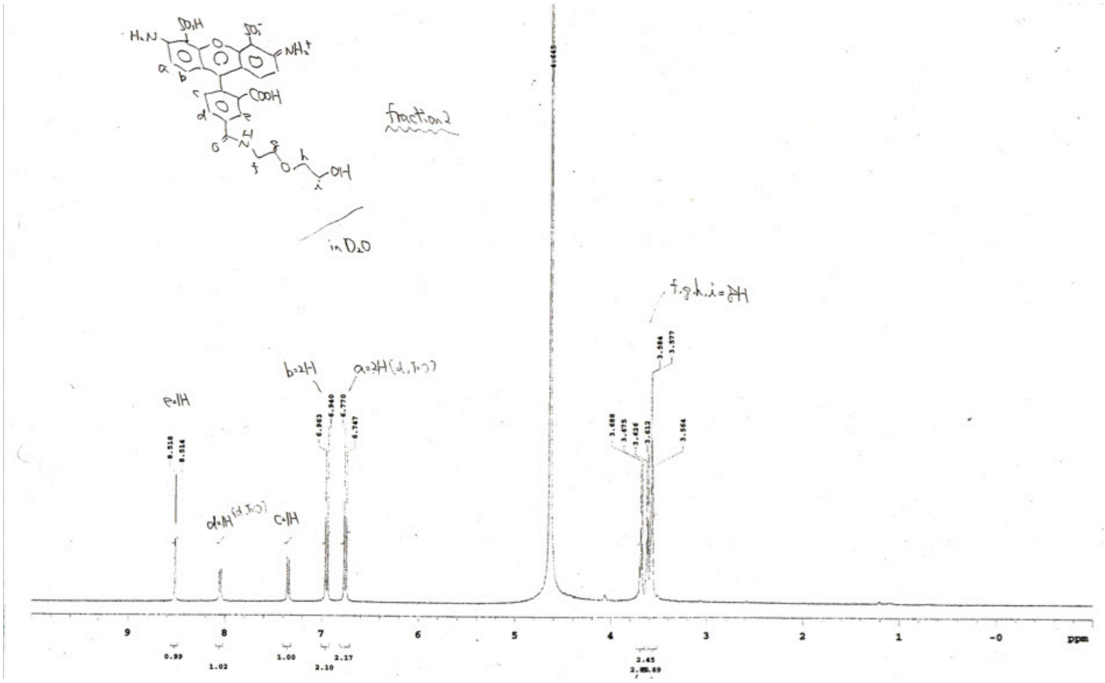

Supplementary Figure 32 | ^{1}H-NMR analysis of Compound 18.

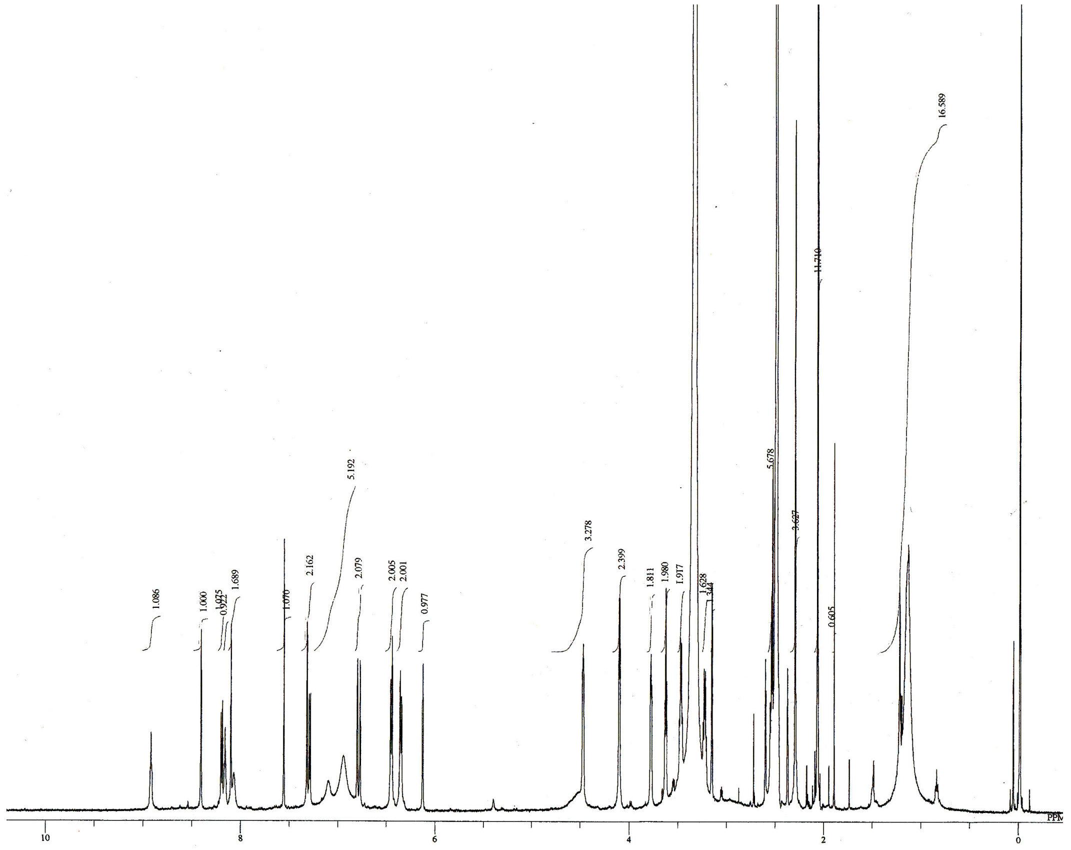

Supplementary Figure 33 | ^{1}H-NMR analysis of CAM2(Ax488).

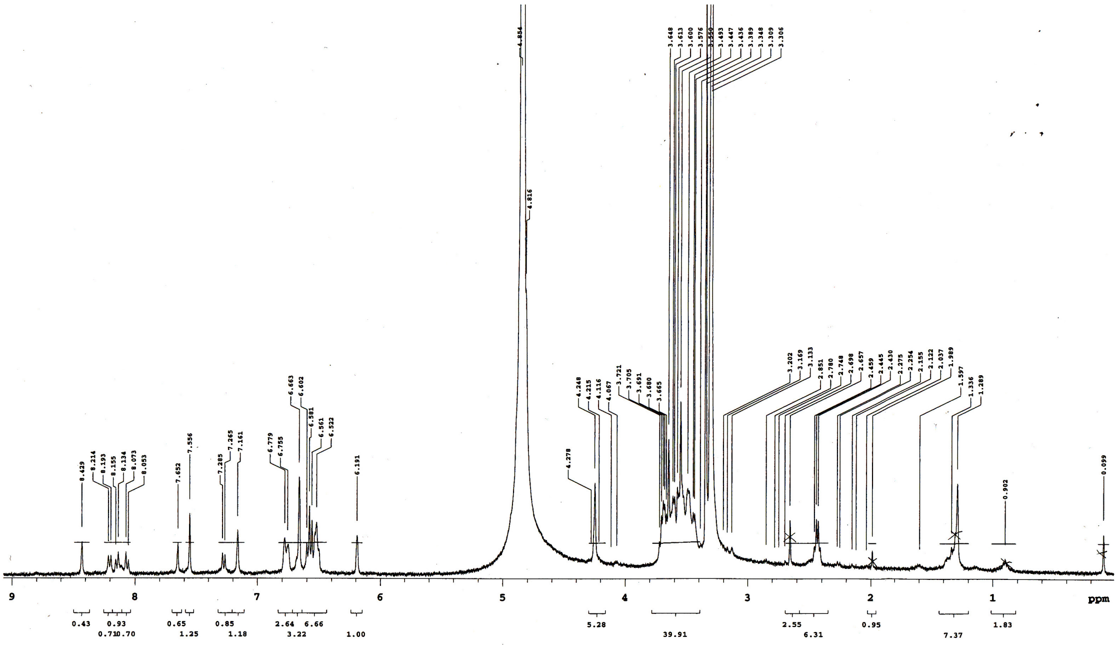

Supplementary Figure 34 | ^{1}H-NMR analysis of Fl-PFQX.

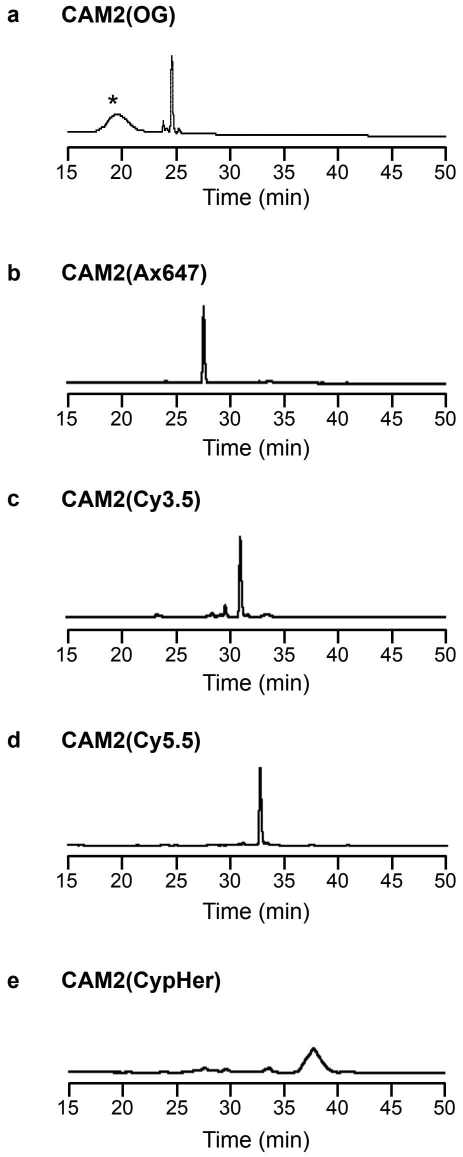

Supplementary Figure 35 | Confirmation of the purity of CAM2 reagents (CAM2(OG), CAM2(Ax647), CAM2(Cy3.5), CAM2(Cy5.5) and CAM2(CypHer)) not characterized by ^{1}H-NMR. (**a**) **CAM2(OG)** was analyzed by RP-HPLC (column; YMC-pack ODS-A, 250 x 25 mm, mobile phase; CH_3CN : 10 mM $AcONH_4$ aq. = 0 : 100 → 40 : 60 (linear gradient over 40 min), flow rate; 10 mL/min, detection; UV (220 nm)). * indicates a noise of the used column. (**b–e**) **CAM2(Ax647), CAM2(Cy3.5) CAM2(Cy5.5) or CAM2(CypHer)** was analyzed by RP-HPLC (column; YMC-Triart C18, 250 x 4.6 mm, mobile phase; CH_3CN : 10 mM $AcONH_4$ aq. = 5 : 95 → 50 : 50 (linear gradient over 50 min), flow rate; 1 mL/min, detection; UV (250 nm)).

Supplementary Table 1 | Averaged characteristics of FRAP analyses for endogenous Fl-AMPARs or exogenously expressed SEP-AMPARs in cultured hippocampal neurons or hippocampal slices.

	Recovery ratio (%)	$t_{1/2}$ (sec) [1)]	Diffusion coefficient ($\mu m^2\ s^{-1}$)
Cultured hippocampal neurons			
Endogenous			
**Fl**-AMPAR (n = 16)	16.2 (± 0.6)	15.6 (± 5.1)	0.090 (± 0.019)
Exogenously expressed			
SEP-AMPAR (n = 8)	54.1 (± 2.0)	95.1 (± 10.1)	– [2)]
Hippocampal slices			
Endogenous			
**Fl**-AMPAR (n = 16)	10.5 (± 0.4)	9.1 (± 2.4)	0.095 (± 0.025)

1) $t_{1/2}$ indicates the half-time of the recovery

2) The value is not determined.

Supplementary Methods

Synthesis and Characterization

General materials and methods for organic synthesis

All chemical reagents and solvents were obtained from commercial suppliers (Aldrich, Tokyo Chemical Industry (TCI), Wako Pure Chemical Industries, Acros Organics, Sasaki Chemical, or Watanabe Chemical Industries) and used without further purification. Thin layer chromatography (TLC) was performed on silica gel 60 F_{254} precoated aluminum sheets (Merck) and visualized by fluorescence quenching or ninhydrin staining. Chromatographic purification was conducted by flash column chromatography on silica gel 60N (neutral, 40–50 μm, Kanto Chemical). ^{1}H-NMR spectra were recorded in deuterated solvents on a Varian Mercury 400 (400 MHz) or JEOL JNM-ECA (600 MHz). ^{13}C-NMR spectra were recorded on 600 MHz JNM-ECA. Chemical shifts were referenced to residual solvent peaks or tetramethylsilane (δ = 0 ppm). Multiplicities are abbreviated as follows: s = singlet, d = doublet, t = triplet, q = quartet, m = multiplet, dd = double doublet, br = broad singlet. High-resolution electrospray ionization quadrupole fourier transform mass spectroscopy (HR-ESI Qq-LTMS) were measured by EXACTIVE mass spectrometer. For NMR analysis of synthesized compounds in this article, see **Supplementary Figures 23–34**. HPLC analysis of **CAM2** reagents not characterized by ^{1}H-NMR is shown in **Supplementary Figure 35**.

Synthesis of **CAM1(OG), CAM2(OG)** and **CAM3(OG)**

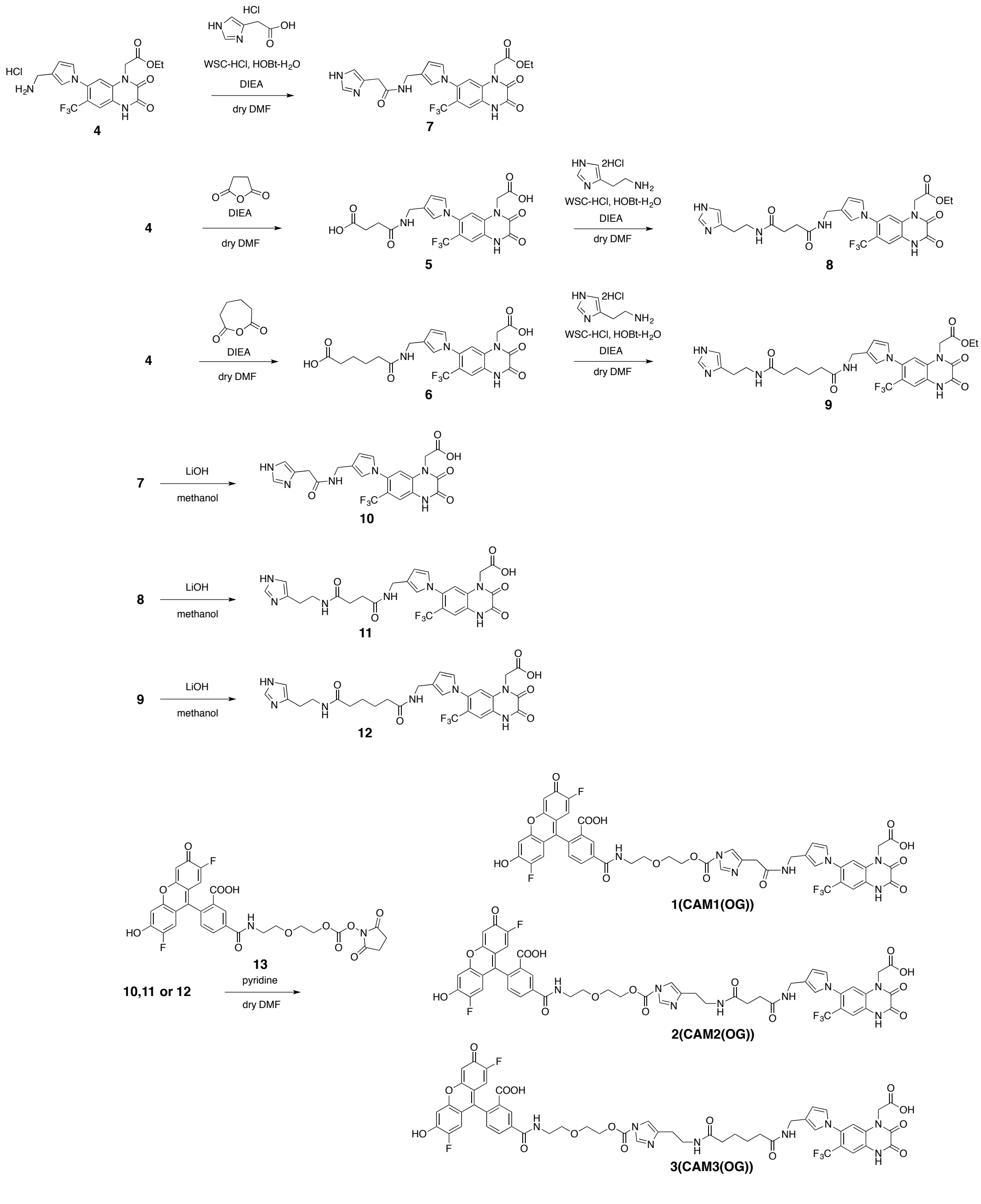

Synthesis of compound **7**

A solution of **4**[2] (70 mg, 160 μmol), 4-imidazole acetic acid hydrochloride (31 mg, 190 μmol), EDCI•HCl (45 mg, 240 μmol), HOBt-H_2O (32 mg, 240 μmol), and DIEA (164 μl, 940 μmol) in dry DMF (2 mL) was stirred for 12 hr at r.t. under N_2 atmosphere. After removal of the solvent by evaporation, the residue was purified by flash column chromatography on silica gel ($CHCl_3$: MeOH = 5 : 1 + 1 % NH_3 aq.) to give **7** (31 mg, 38 % yield) as a white solid. ^{1}H-NMR (400 MHz, CD_3OD) δ 7.60 (s, 2H), 7.23 (s, 1H), 6.98 (s, 1H), 6.78 (m, 2H), 6.21 (s, 1H), 5.03 (s, 2H), 4.22 (m, 4H), 3.51 (s, 2H), 1.23 (t, *J* = 7.2 Hz, 3H).

Synthesis of compound **5**

A solution of **4** (166 mg, 370 μmol), succinic anhydride (45 mg, 450 μmol) and DIEA (130 μl, 0.74 mmol) in dry DMF (5 mL) was stirred for 2.5 h at r.t. under N_2 atmosphere. After removal of the solvent by evaporation, the residue was washed by water to give **5** (92 mg, 49 % yield) as a white solid. ^{1}H-NMR (400 MHz, CD_3OD) δ 7.60 (s, 1H) 7.28 (s, 1 H), 6.81 (d, *J* = 7.2 Hz, 2H), 6.23 (s, 1H), 5.01 (s, 2H), 4.23 (m, 4H), 2.50 (m, 4H), 1.24 (d, *J* = 5.2 Hz, 3 H).

Synthesis of compound **8**

A solution of **5** (92 mg, 180 μmol), EDCI•HCl (45 mg, 240 μmol), HOBt-H_2O (32 mg, 240 μmol), histamine•2HCl (43 mg, 240 μmol), and DIEA (158 μl, 910 μmol) was stirred for 3 h at r.t. under N_2 atmosphere. After removal of the solvent by evaporation, the residue was purified by flash column chromatography on silica gel ($CHCl_3$: MeOH = 1 : 5 + 1 % NH_3 aq.) to give **8** (72 mg, 67 % yield) as a white solid. ^{1}H-NMR (400 MHz, CD_3OD) δ 7.59 (s, 1H), 7.55 (s, 1H), 7.24 (s, 1H), 6.81 (m, 3H), 6.21 (t, *J* = 4.2 Hz, 1H), 5.02 (s, 3H), 4.24 (m, 4H), 2.60 (m, 8H), 1.25 (t, *J* = 5.4 Hz, 3H).

Synthesis of compound **6**

A solution of **4** (130 mg, 290 μmol), adipic anhydride (45 mg, 0.45 μmol), and DIEA (102 μl, 580 μmol) in dry DMF (1 mL) was stirred for 3 h at r.t. under N_2 atmosphere. After removal of the solvent by evaporation, the residue was purified by column chromatography on silica gel ($CHCl_3$: MeOH = 10 : 1 + 0.1 % AcOH) to give **6** (137

mg, 86 % yield) as a white solid. ^{1}H-NMR (400 MHz, CD_3OD) δ 7.90 (s, 1H), 7.28 (s, 1H), 7.24 (s, 1H), 6.77 (m, 2H), 6.20 (d, *J* = 7.2 Hz, 1H), 5.03 (s, 2H), 4.21 (m, 4H), 2.30 (m, 4H), 1.63 (m, 4H), 1.24 (t, *J* = 7.2 Hz, 3H).

Synthesis of compound **9**

A solution of **6** (137 mg, 250 μmol), EDCI•HCl (63 mg, 330 μmol), HOBt-H_2O (45 mg, 330 μmol), histamine•2HCl (61 mg, 330 μmol), and DIEA (221 μl, 1.27 mmol) in dry DMF (5 mL) was stirred for 2 h at r.t. under N_2 atmosphere. After removal of the solvent by evaporation, the residue was purified by column chromatography on silica gel ($CHCl_3$: MeOH = 5 : 1 + 1 % NH_3 aq.) to give crude **9** (33 mg, 0.12 mmol), and used for the next step without further purification.

Synthesis of compound **CAM1(OG)**

A solution of **7** (20 mg, 33 μmol) and 0.5 M LiOH aq. (284 μl, 142 μmol) in MeOH (0.5 mL) was stirred for 3 h at r.t. After neutralization with 1 N HCl and removal of the solvent, **10** was obtained as a crude, and used for the next step without further purification.

A solution of **10**, **13**[3] **(**25 mg, 39 μmol), and pyridine (11.4 μl, 140 μmol) in dry DMF (1 mL) was stirred for 6 h at r.t. The mixture was purified by RP-HPLC (column; YMC-pack ODS-A, 250 x 25 mm, mobile phase; CH_3CN : 10 mM $AcONH_4$ aq. = 0 : 100 → 30 : 70 (linear gradient over 40 min), flow rate; 10 mL/min, detection; UV (220 nm)) to give **CAM1(OG) (**2.8 mg, 8.3 % yield in 2 steps) as an orange solid. HR-ESI MS *m/e* calcd for $[M-H]^-$ 1014.2000, found 1014.2008.

Synthesis of compound **CAM2(OG)**

A solution of **8** (20 mg, 33 μmol) and 0.5 M LiOH aq. (165 μl, 83 μmol) in MeOH (0.5 mL) was stirred for 3 h at r.t. After neutralization with 1 N HCl and removal of solvent, **11** was obtained as a crude, and used for the next step without further purification.

A solution of **11**, **13** (21 mg, 33 μmol), and pyridine (10.6 μl, 130 μmol) in dry DMF (0.5 mL) was stirred for 6 h at r.t. The mixture was purified by RP-HPLC (column; YMC-pack ODS-A, 250 x 25 mm, mobile phase; CH_3CN : 10 mM $AcONH_4$ aq. = 0 :

100 → 40 : 60 (linear gradient over 40 min), flow rate; 10 mL/min, detection; UV (220 nm)) to give **CAM2(OG)** (1.2 mg, 3.3 % yield in 2 steps) as an orange solid. HR-ESI MS *m/e* calcd for $[M+Na]^+$ 1123.2504, found 1123.2507. In the ^{1}H-NMR analysis in CD_3OD or DMSO-d_6, we could not assign the peak due to the peak broadening (see **Supplementary Figure 27** for ^{1}H-NMR in DMSO-d_6).

Synthesis of compound **CAM3(OG)**

A solution of crude **9** (33 mg) and 0.5 M LiOH aq. (210 µl, 105 µmol) in MeOH (0.5 mL) was stirred for 5 h at r.t. After removal of the solvent, the residue was purified by RP-HPLC (column; YMC-pack ODS-A, 250 x 25 mm, mobile phase; CH_3CN (containing 0.1 % TFA) : H_2O (containing 0.1 % TFA) = 0 : 100 → 35 : 65 (linear gradient over 30 min), flow rate; 10 mL/min, detection; UV (220 nm)) to give **12** (18 mg, 26 µmol) .

A solution of **12** (18 mg, 26 µmol), **13** (17 mg, 26 µmol), and pyridine (10.6 µl, 130 µmol) in dry DMF (0.5 mL) was stirred for 6 h at r.t. The mixture was purified by RP-HPLC (column; YMC-pack ODS-A, 250 x 25 mm, mobile phase; CH_3CN : 10 mM $AcONH_4$ aq. = 0 : 100 → 30 : 70 (linear gradient over 40 min), flow rate; 10 mL/min, detection; UV (220 nm)) to give **CAM3(OG)** (1 mg, 3.4 % yield) as an orange solid. HR-ESI MS *m/e* calcd for $[M+H]^+$ 1129.2997, found 1129.3009.

Synthesis of **CAM2(Fl)**, **CAM2(Bt)**, **CAM2(Ax647)**, **CAM2(CypHer)**, **CAM2(Cy3.5)** and **CAM2(Cy5.5)**

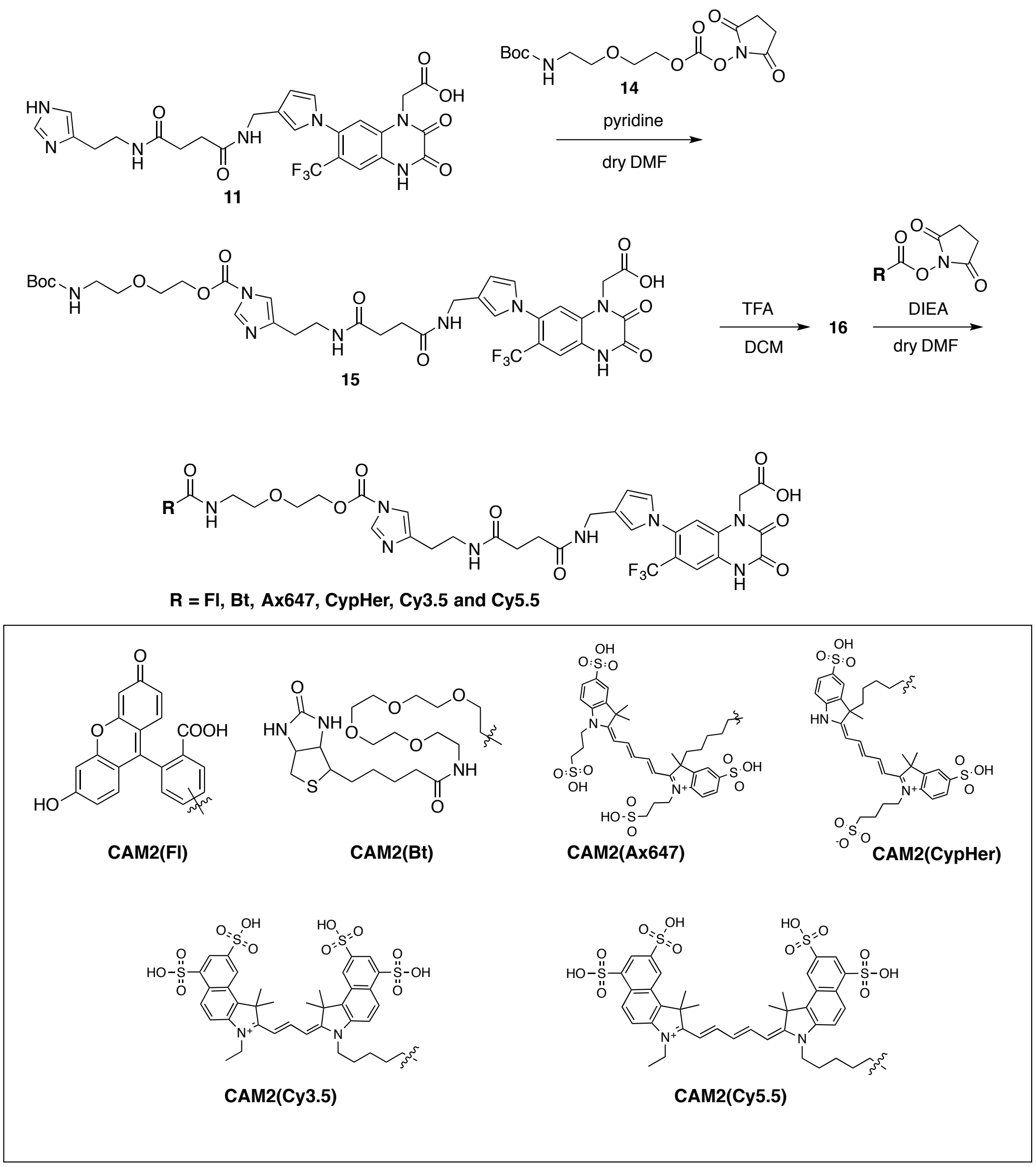

Synthesis of compound **15**

A solution of crude **11** (15 μmol), **14**[4] (15.6 mg, 45 μmol), and pyridine (10 μL, 120 μmol) in DMF (0.5 mL) was stirred for 12 hr at r.t. under N_2 atmosphere. The crude solution was purified by RP-HPLC (column; YMC-pack ODS-A, 250 x 25 mm, mobile phase; CH_3CN : 10 mM $AcONH_4$ aq. = 0 : 100 → 50 : 50 (linear gradient over 45 min), flow rate; 10 mL/min, detection; UV (220 nm) to give **15** (5.7 mg, 47 %

yield) as a white solid. MALDI-TOF MS (CHCA) *m/z* calcd for $[M+H]^+$ 807.2920, found 807.326

Synthesis of compound **CAM2(Fl)**

A solution of **15** (1 mg, 1.4 μmol) in CH_2Cl_2 (1.5 mL) / TFA (0.5 mL) was stirred for 5 h at r.t. After removal of the solvent by evaporation, the residual TFA was further removed azeotropically with toluene (x2) to give Boc-deprotected **16**. The compound was used for the next step without further purification.

A solution of **16**, 5,6-carboxyfluorescein succinimidyl ester (1.4 mg, 3.0 μmol), and DIEA (1.2 μL, 6.8 μmol) in dry DMF (0.5 mL) was stirred for 2 h at r.t. under N_2 atmosphere. After removal of the solvent by evaporation, the residue was purified by RP-HPLC (column; YMC-pack ODS-A, 250 x 25 mm, mobile phase; CH_3CN : 10 mM $AcONH_4$ aq. = 5 : 95 → 35 : 65 (linear gradient over 60 min), flow rate; 10 mL/min, detection; UV (220 nm)) to give **CAM2(Fl)** (0.4 mg, 28 % yield in 2 steps) as a orange solid. HR-ESI MS *m/z* calcd for $[M+H]^+$ 1065.2873, found 1065.2853 ^{1}H-NMR (600 MHz, DMSO-d_6) δ 8.89 (t, *J* = 5.4 Hz, 1H), 8.41 (s, 1H), 8.19 (d, *J* = 7.8, 1H), 8.11 (t, *J* = 5.4 Hz, 1H), 7.54 (s, 1H), 7.29 (s, 1H), 7.02 (s, 1H), 6.76 (s, 1H), 6.74 (s, 1H), 6.66 (d, *J* = 1.8 Hz, 2H), 6.55 (s, 1H), 6.53 (s, 2H), 6.51 (d, *J* = 2.4 Hz, 2H), 6.50 (d, *J* = 2.4, 1H), 6.09(m, 1H), 4.48(m, 4H), 4.08 (d, *J* = 5.4 Hz, 2H), 3.78 (m, 2H), 3.64 (t, *J* = 6.0, 2H), 3.48 (m, 2H), 3.22 (m, 2H), 2.52 (m, 2H), 2.29 (m, 4H) ^{13}C-NMR (150 MHz, DMSO-d_6) δ 171.27 (s, 1C), 171.10 (s, 1C), 168.11 (s, 1C), 167.71 (s, 1C), 164.75 (s, 1C), 159.84 (s, 1C), 154.71 (s, 1C), 154.57 (s, 1C), 153.33 (s, 1C), 151.77 (s, 1C), 148.23 (s, 1C), 141.20 (s, 1C), 136.58 (s, 1C), 136.07 (s, 1C), 134.46 (s, 1C), 133.312 (s, 1C), 130.46 (s, 1C), 128.94 (s, 1C), 126.47 (s, 1C), 124.75 (s, 1C), 124.08 (s, 1C), 123.54 (s, 1C), 123.23 (s, 1C), 122.45 (s, 1C), 121.10 (s, 1C), 118.75 (q, *J* = 119 Hz, 1C), 116.48, 113.45 (d, *J* = 27 Hz, 1C), 113.36 (s, 1C), 112.66 (s, 1C), 109.20 (s, 1C), 108.83 (s, 1C), 102.24 (s, 1C), 68.69 (s, 1C), 67.53 (s, 1C), 67.01 (s, 1C), 46.85 (s, 1C), 37.84 (s, 1C), 35.28 (s, 1C), 31.09 (s, 1C), 30.96 (s, 1C), 27.82 (s, 1C).

Synthesis of compound **CAM2(Bt)**

A solution of **16** (1.2 μmol), **NHS-PEG4-Biotin** (2 mg, 3.4 μmol) and DIEA (1.1 μL, 6.2 μmol) in dry DMF (0.5 mL) was stirred for 1 h at r.t. under N_2 atmosphere. After removal of the solvent by evaporation, the residue was purified by RP-HPLC (column; YMC-pack ODS-A, 250 x 25 mm, mobile phase; CH_3CN : 10 mM $AcONH_4$ aq. = 5 : 95 → 50 : 50 (linear gradient over 70 min), flow rate; 10 mL/min, detection; UV (220 nm)) to give **CAM2(Bt)** (0.6 mg, 41 % yield) as a white solid. HR-ESI MS *m/z* calcd for $[M+H]^+$ 1180.4591, found 1180.4557 ^{1}H-NMR (600 MHz, CD_3OD) δ 8.55 (t, *J* = 5.4 Hz, 1H), 8.46–8.34 (m, 4H), 8.33 (d, *J* = 1.2 Hz, 1H), 7.56 (s, 1H), 7.44 (s, 1H), 7.13 (s, 1H), 6.85 (br, 1H), 6.82 (br, 1H), 6.21 (t, *J* = 2.4 Hz, 1H), 4.56–4.55 (m, 2H), 4.50–4.48 (m, 1H), 4.31–4.29 (m, 1H), 4.25 (s, 2H), 3.81–3.80 (m, 2H), 3.68 (t, *J* = 6.0 Hz, 2H), 3.64–3.56 (m, 14H), 3.51 (t, *J* = 5.4 Hz, 2H), 3.41 (t, 7.2 Hz, 2H), 3.37–3.34 (m, 4H), 3.21–3.18 (m, 1H), 2.93–2.90 (m, 1H), 2.72–2.68 (m, 3H), 2.49–2.47 (m, 4H), 2.43 (t, *J* = 6.0 Hz, 2H), 2.21 (t, *J* = 7.2 Hz, 2H), 1.74–1.55 (m, 4H), 1.41–1.40 (m, 2H)

Synthesis of compound **CAM2(Ax647)**

A solution of **16** (1.2 μmol), Alexa647 succinimidyl ester (2 mg, 1.6 μmol) and DIEA (10 μL, 57 μmol) in dry DMF (0.5 mL) was stirred for 5 h at r.t. under N_2 atmosphere. After removal of the solvent by evaporation, the residue was purified by RP-HPLC (column; YMC-pack ODS-A, 250 x 25 mm, mobile phase; CH_3CN : 10 mM $AcONH_4$ aq. = 5 : 95 → 50 : 50 (linear gradient over 60 min), flow rate; 10 mL/min, detection; UV (220 nm)) to give **CAM2(Ax647)** (1.4 mg, 77 % yield) as a blue solid. HR-ESI MS *m/z* calcd for $[M]^+$ 1547.4122, found 1547.4153

Synthesis of compound **CAM2(CypHer)**

A solution of **16** (2 μmol), CypHer5E succinimidyl ester (2 mg, 2 μmol) and DIEA (2 μL, 12 μmol) in dry DMF (0.5 mL) was stirred for 18 h at r.t. under N_2 atmosphere. After removal of the solvent by evaporation, the residue was purified by RP-HPLC (column; YMC-pack ODS-A, 250 x 25 mm, mobile phase; CH_3CN : 10 mM $AcONH_4$ aq. = 5 : 95 → 30 : 70 (linear gradient over 55 min), flow rate; 10 mL/min, detection; UV (220 nm)) to give **CAM2(CypHer)** (0.6 mg, 21%) as a blue solid. HR-ESI MS *m/z* calcd for $[M+H]^+$ 1439.4240, found 1439.4249

Synthesis of compound **CAM2(Cy3.5)**

A solution of **16** (0.51 μmol), Cy3.5succinimidyl ester (1 mg, 0.77 μmol) and DIEA (10 μL, 57 μmol) in dry DMF (0.5 mL) was stirred for 5 h at r.t. under N_2 atmosphere. After removal of the solvent by evaporation, the residue was purified by RP-HPLC (column; YMC-pack ODS-A, 250 x 25 mm, mobile phase; CH_3CN : 10 mM $AcONH_4$ aq. = 5 : 95 → 50 : 50 (linear gradient over 60 min), flow rate; 10 mL/min, detection; UV (220 nm)) to give **CAM2(Cy3.5)** (0.3 mg, 59 % yield) as a purple solid. HR-ESI MS *m/z* calcd for $[M-3H]^{2-}$ 788.1795 found 788.1796

Synthesis of compound **CAM2(Cy5.5)**

A solution of **16** (0.51 μmol), Cy5.5succinimidyl ester (1 mg, 0.76 μmol) and DIEA (10 μL, 57 μmol) in dry DMF (0.5 mL) was stirred for 5 h at r.t. under N_2 atmosphere. After removal of the solvent by evaporation, the residue was purified by RP-HPLC (column; YMC-pack ODS-A, 250 x 25 mm, mobile phase; CH_3CN : 10 mM $AcONH_4$ aq. = 5 : 95 → 50 : 50 (linear gradient over 60 min), flow rate; 10 mL/min, detection; UV (220 nm)) to give **CAM2(Cy5.5)** (0.6 mg, 73% yield) as a blue solid. HR-ESI MS *m/z* calcd for $[M-3H]^{2-}$ 801.1873 found 801.1855

Synthesis of **CAM2(Ax488)**

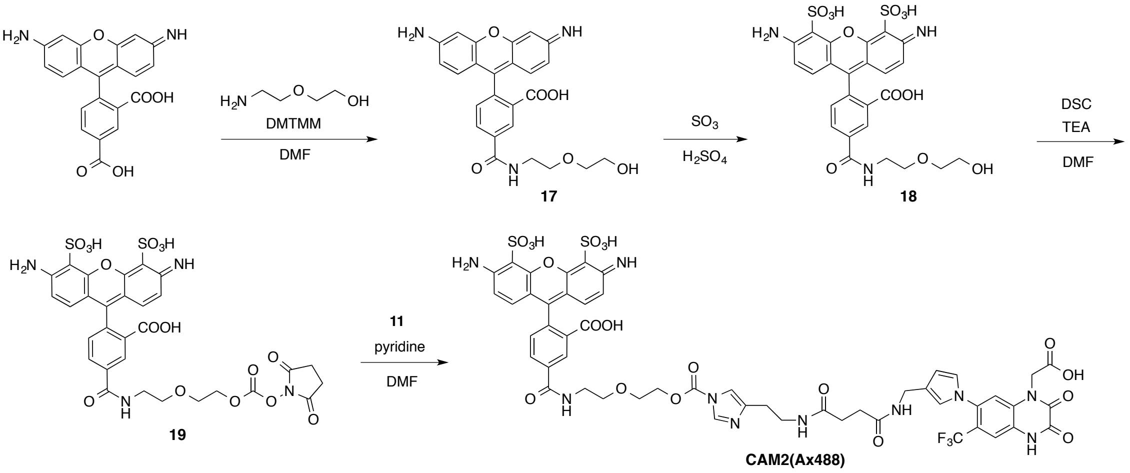

Synthesis of compound **17**

A solution of 5-carboxy rhodamine green (50 mg, 130 μmol), 4-(4,6-Dimethoxy-1,3,5-triazin-2-yl)-4-methylmorpholinium Chloride (72 mg, 260 μmol), and 2-(2-aminoethoxy)ethanol (41 mg, 390 μmol) in dry DMF (1.5 ml) was stirred for 4h at r.t. under N_2 atmosphere. After removal of the solvent by evaporation, the residue was recrystallized from hot methanol (50 mL) to give **17** (40 mg, 65 % yield) as a yellow solid. ^{1}H-NMR (400 MHz, DMSO-d_6) δ 8.83 (br, 1H), 8.39 (s, 1H), 8.20 (d, J = 8.0 Hz, 1H), 7.30 (d, J = 8.0 Hz, 1H), 6.31 (m, 6H), 5.60 (s, 4H), 4.58 (t, J = 5.6 Hz, 1H), 3.47 (m, 8H).

Synthesis of compound **18**

A solution of **17** (32 mg, 69 μmol) in 30 % fuming H_2SO_4 (1 mL) was stirred for 12 h on ice. Dry dioxane (5 mL) and Et_2O (10 mL) were poured to the solution and the resulting supernatant was removed completely and the residue was dried in *vacuo*. The crude mixture was purified by RP-HPLC (column; YMC-pack ODS-A, 250 x 25 mm, mobile phase; CH_3CN (containing 0.1 % TFA) : H_2O (containing 0.1 % TFA) = 0 : 100 → 20 : 80 (linear gradient over 40 min), flow rate; 10 mL/min, detection; UV (220 nm)) to give **18** (10 mg, 21 % yield) as a yellow solid. ^{1}H-NMR (400 MHz, D_2O) δ 8.52 (s, 1H), 8.05 (d, J = 8.0 Hz, 1H), 7.38 (d, J = 8.0 Hz, 1H), 6.95 (d, J = 9.2 Hz, 2H), 6.76 (d, J = 9.2 Hz, 2H), 3.61 (m, 8H).

Synthesis of compound **19**

A solution of **18** (7.0 mg, 10 μmol), *N,N'*-disuccimidyl carbonate (6.4 mg, 25 μmol), and triethylamine (3.5 μl, 25 μmol) in dry DMF was stirred for 6 h at r.t. under N_2 atmosphere. After removal of the solvent by evaporation, the residue was precipitated by CH_2Cl_2 (2 mL) and Et_2O (3 mL) to give **19** (10 mg) containing some impurities, which was used for the next step without further purification.

Synthesis of compound **CAM2(Ax488)**

A solution of **19** (10 mg), **11** (15.6 μmol) and pyridine (6.0 μl, 74 μmol) in dry DMF (0.5 mL) was stirred for 12 h at r.t. under N_2 atmosphere. After removal of the solvent by evaporation, the residue was purified by RP-HPLC (column; YMC-pack ODS-A, 250 x 25 mm, mobile phase; CH_3CN : 10 mM $AcONH_4$ aq. = 0 : 100 → 40 : 60 (linear gradient over 55 min), flow rate; 10 mL/min, detection; UV (220 nm)) to give **CAM2(Ax488)** (2.0 mg, 10 % yield) as an orange solid. HR-ESI MS *m/z* calcd for $[M-H]^-$ 1221.2172, found 1221.2230 ^{1}H-NMR (600 MHz, DMSO-d_6) δ 8.92 (t, *J* = 4.8 Hz, 1H), 8.41 (s, 1H), 8.19 (d, *J* = 7.8 Hz, 1H), 8.16 (t, *J* = 6.0 Hz, 1H), 8.10 (s, 1H), 7.56 (s, 1H), 7.32 (s, 1H), 7.29 (d, *J* = 7.8 Hz, 1H), 7.10 (br, 1H), 6.94 (br, 4H), 6.80 (s, 1H), 6.77 (s, 1H), 6.45 (d, 8.4 Hz, 2H), 6.35 (d, 8.4 Hz, 2H), 6.13 (s, 1H), 4.48 (t, *J* = 4.2 Hz, 2H), 4.11 (d, *J* = 5.4 Hz, 2H), 3.78 (t, *J* = 4.2 Hz, 2H), 3.63 (t, *J* = 6.0 Hz, 2H), 3.49–3.46 (m, 2H), 3.24–3.21 (m, 2H), 3.15 (s, 1H), 2.55–2.51 (m, 2H), 2.30 (s, 4H)

Synthesis of **Fl-PFQX**

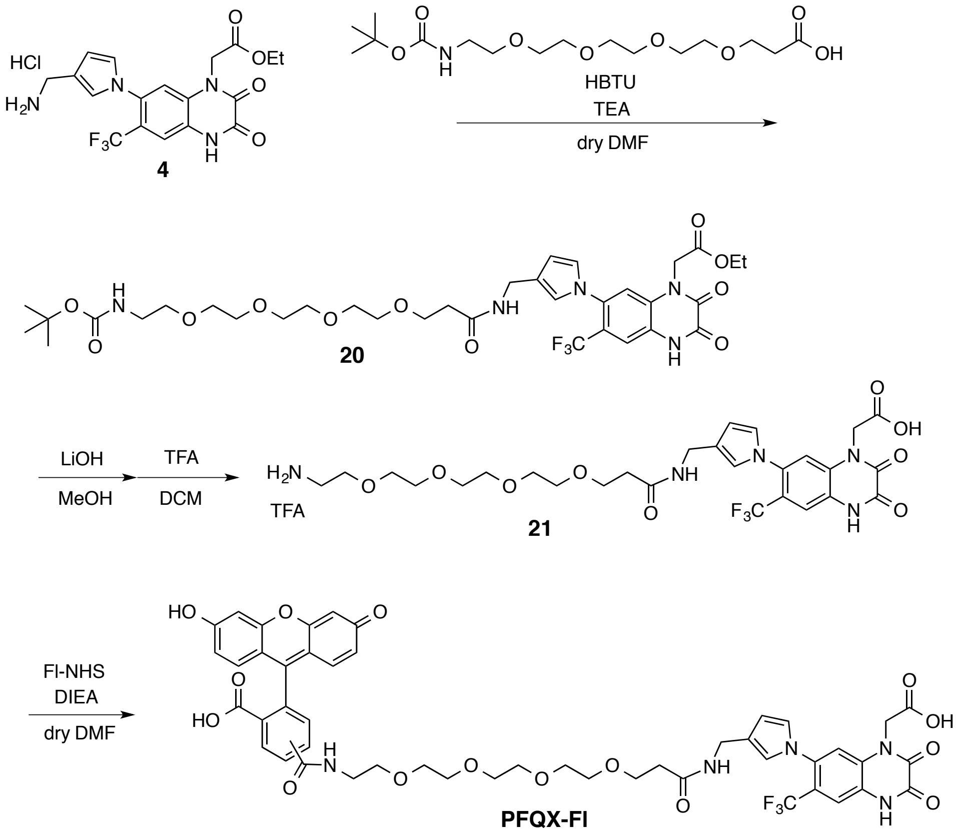

Synthesis of compound **20**

A solution of **4** (20 mg, 45 μmol), Boc-(PEG)$_3$-COOH (19 mg, 51 μmol), HBTU (20 mg, 54 μmol), and TEA (31 μl, 220 μmol) in dry DMF (1 mL) was stirred for 3 hr at r.t. under N_2 atmosphere. After removal of the solvent by evaporation, the residue was purified by flash column chromatography on silica gel ($CHCl_3$: MeOH = 20 : 1) to give crude **20** (22 mg) and used for the next step without further purification.

Synthesis of compound **21**

A solution of crude **20** (10 mg) and 0.5 N LiOH (130 μl, 66 μmol) in MeOH (1 mL) was stirred for 12 hr at r.t. After removal of solvent, DCM (1 mL), and TFA (1 mL) were added and stirred for 12 hr at r.t. After removal of the solvent by evaporation, the residual TFA was further removed azeotropically with toluene (x2) to give crude **21**. The compound was used for the next step without further purification.

Synthesis of compound **PFQX-Fl**

A solution of crude **21** (10 mg) and 5, 6-carboxyfluorescein succinimidyl ester (9.4mg, 20 μmol), and DIEA (23 μl, 130 μmol) in dry DMF (0.5 mL) was stirred for 5 hr at r.t. under N_2 atomosphere. The mixture was purified by RP-HPLC (column; YMC-pack ODS-A, 250 x 25 mm, mobile phase; CH_3CN (0.1 % TFA) : H_2O (0.1 % TFA) = 5 : 95 → 50 : 50 (linear gradient over 60 min), flow rate; 10 mL/min, detection; UV (220 nm)) to give **PFQX-Fl (**3 mg, 6.7 % yield in 3 steps) as a yellow solid. HR-ESI MS *m/z* calcd for $[M+H]^+$ 988.2865, found 988.2859 ^1H-NMR (400 MHz, CD_3OD) δ 8.43 (s, 0.5H), 8.20 (d, *J* = 8.0 Hz, 0.5H), 8.14 (d, *J* = 8.0 Hz, 0.5H), 8.06 (d, *J* = 8.0 Hz, 0.5H), 7.65 (s, 0.5H), 7.56 (s, 1H), 7.27 (d, *J* = 8.0 Hz, 0.5H), 7.16 (s, 1H) 6.78–6.76 (m, 2H), 6.66 (s, 2H), 6.60–6.52 (m, 4H), 6.19 (s, 1H), 4.25 (s, 2H), 3.72–3.39 (m, 18H), 2.46–2.42 (m, 2H)

Supplementary Reference

1. Coleman, S. K. *et al.* Aggregation Limits Surface Expression of Homomeric GluA3 Receptors. *J. Biol. Chem.* **291**, 8784-8794 (2016)
2. Lubisch, W., Behl, B. & Hofmann, H.P. Pyrrolylquinoxalinediones : A new class of AMPA receptor antagonists. *Bioorg. Med. Chem. Lett.* **6**, 2887–2892 (1996)
3. Miki, T. *et al*. LDAI-Based Chemical Labeling of Intact Membrane Proteins and Its Pulse-Chase Analysis under Live Cell Conditions. *Chem. Biol.* **21**, 1013–1022 (2014)
4. Fujishima, S., Yasui, R., Miki, T., Ojida, A. & Hamachi, I. Ligand-Directed Acyl Imidazole Chemistry for Labeling of Membrane-Bound Proteins on Live Cells. *J. Am. Chem. Soc.* **134**, 3961–3964 (2012)
